# Multi-model genome-wide association studies for appearance quality in rice

**DOI:** 10.3389/fpls.2023.1304388

**Published:** 2024-01-11

**Authors:** Supriya Sachdeva, Rakesh Singh, Avantika Maurya, Vikas Kumar Singh, Uma Maheshwar Singh, Arvind Kumar, Gyanendra Pratap Singh

**Affiliations:** ^1^ Division of Genomic Resources, ICAR-National Bureau of Plant Genetic Resources (NBPGR), New Delhi, India; ^2^ International Rice Research Institute, South Asia Hub, International Crop Reseach Institute for Semi Arid Tropics (ICRISAT), Hyderabad, India; ^3^ International Rice Research Institute, South Asia Regional Centre (ISARC), Varanasi, India; ^4^ International Crops Research Institute for the Semi-Arid Tropics, Patancheru, Telangana, India; ^5^ Indian Council of Agricultural Research (ICAR)-National Bureau of Plant Genetic Resources, New Delhi, India

**Keywords:** rice, grain quality, QTNs, candidate genes, GWAS

## Abstract

Improving the quality of the appearance of rice is critical to meet market acceptance. Mining putative quality-related genes has been geared towards the development of effective breeding approaches for rice. In the present study, two SL-GWAS (CMLM and MLM) and three ML-GWAS (FASTmrEMMA, mrMLM, and FASTmrMLM) genome-wide association studies were conducted in a subset of 3K-RGP consisting of 198 rice accessions with 553,831 SNP markers. A total of 594 SNP markers were identified using the mixed linear model method for grain quality traits. Additionally, 70 quantitative trait nucleotides (QTNs) detected by the ML-GWAS models were strongly associated with grain aroma (AR), head rice recovery (HRR, %), and percentage of grains with chalkiness (PGC, %). Finally, 39 QTNs were identified using single- and multi-locus GWAS methods. Among the 39 reliable QTNs, 20 novel QTNs were identified for the above-mentioned three quality-related traits. Based on annotation and previous studies, four functional candidate genes (*LOC_Os01g66110*, *LOC_Os01g66140*, *LOC_Os07g44910*, and *LOC_Os02g14120*) were found to influence AR, HRR (%), and PGC (%), which could be utilized in rice breeding to improve grain quality traits.

## Introduction

Cultivated rice (*Oryza sativa* L.) is an important source of calories for more than half of the global population. With improved living standards and increasing awareness among people worldwide, there is a growing demand for the consumption of superior quality healthier rice varieties (Bao, 2014; Adjah et al., 2020; Selvaraj et al., 2021; Hori and Sun, 2022). Therefore, high-quality rice has become a paramount consideration for rice breeders, consumers, and producers (Qiu et al., 2021). The crucial determinants of rice grain quality include appearance, milling, nutritional composition, aroma, and cooking properties. Recently, more efforts have been made to breed rice varieties with desirable traits in terms of higher head rice recovery (HRR, %), and lower percentage of chalky grains (PGC, %) by discovering key haplotype variations, thereby harnessing allelic diversity in the germplasm (Selvaraj et al., 2021). Currently, molecular advances and genome sequencing platforms with lower costs have aided in cloning and functionally dissecting a series of genetic factors/quantitative trait loci (QTLs) in rice (Varshney et al., 2014; Abbai et al., 2019). Genetic studies have shown that multiple factors control each quality trait reflecting the intricate nature of the rice quality traits (Li et al., 2022). The genes affecting these physicochemical characteristics are related to starch biosynthesis, the metabolism of seed storage proteins (SSPs), and specific nutraceutical compounds (Biselli et al., 2015). Grain chalkiness, for example, is associated with many genes such as *Flo2*, *Chalk5*, *GIF2*, *LTPs*, *GBSS I*, *OsPUL*, *OsBT1*, *OsBE1*, and *SSIIa* (Li et al., 2014a; Wang et al., 2018), and several QTLs have been detected and widely distributed across the rice genome (Zhang H. et al., 2019; Hori et al., 2021), two of which have been fine-mapped by association and linkage mapping, such as qPGWC-7 (Zhou et al., 2009), qPGWC-8 (Guo et al., 2011; Zhao et al., 2016), and one QTL cluster mapped on chromosome 4 by single and joint mapping studies between the markers id4007289 and RM252. Loss of function mutations and genic interactions between the alleles of well-known genes responsible for biosynthesis of starch, *viz.*, *GBSSI*, *SS2a*, *SS3a*, *SS4b*, *BE2b*, and, *ISA1* gene have been shown to increase the amount of resistant starch in rice, which is believed to be crucial for improving human health (Zhang C. et al., 2019; Fujita et al., 2022; Miura et al., 2022).

Regarding the percentage of rice recovery determining rice grain quality, approximately 34 genes/QTLs have been documented in all rice chromosomes, which are largely influenced by the environment (Bao, 2014). A common QTL for grain size and head rice recovery was also detected on chromosome 3, suggesting a relationship between these two traits at the genetic level (Tan et al., 2001). An increase in grain yield has been reported in near-isogenic lines (NILs) introduced with the null allele of rice chalkiness gene *PDIL1-1*, explaining significant differences in phenotype between the genetic makeup of the rice cultivars; however, there was an increase in grain chalkiness (Hori and Sun, 2022). The appearance and rice grain quality are closely related to its rice grain size (Xie et al., 2013; Bao, 2019). Interestingly, pleiotropic effects have been reported in 25 cloned QTLs identified for multiple grain size-controlled traits, namely rice yield, appearance, and grain quality (Wang et al., 2018). Furthermore, the *gw2* WY3 allele had positive effects on grain yield, but reduced grain quality by increasing PGC (%) and decreasing HRR (%) (Song et al., 2007).

Fragrant rice is a special group with a distinct aroma, flavor, and medicinal, antioxidant, and stress-resistance properties. To date, more than 200 aroma compounds have been documented in fragrant rice (Champagne, 2008) and 2-acetly-1-pyrroline (2-AP) has been recognized as the most prominent compound contributing to aroma production in rice (Poonlaphdecha et al., 2016; Wakte et al., 2017) which is under the control of a recessive gene *Badh2*. RNA Seq studies have shown that the expression of heavy metal transporters in response to zinc at the transcriptional and post-transcriptional levels, and their epigenetic modifications, regulate the biosynthesis of 2-AP in aromatic rice varieties (Imran et al., 2022). The haplotype diversity of the *Badh2* gene was investigated in 22 fragrant landraces from Thailand, identifying four new haplotypes (H1, H2, H3, and H4). These *badh2* alleles may serve as functional markers, and landraces with a favorable haplotype (H1) could be employed as genetic resources in rice breeding programs (Chan-In et al., 2020). Several other genes affecting seed development and quality traits have been characterized, such as *GW2* (Song et al., 2007), *GS3* (Sun et al., 2018), *GS2* (Hu et al., 2015), *GS5* (Xu et al., 2015), *GS9* (Zhao et al., 2018), *GW5* (Duan et al., 2017), *GLW7* (Si et al., 2016), and *OsMAPK6* (Liu et al., 2015). Therefore, understanding the molecular basis of these traits is a prerequisite for identifying novel alleles and donors related to high grain quality, which could considerably improve rice breeding efficiency (Yano et al., 2016; Wang et al., 2017; Abbai et al., 2019; Misra et al., 2019; Verma et al., 2021; Zhong et al., 2021). These newly recognized superior versions of quality genes might then be taken together through the rapid and undoubtedly proved concept of ‘haplotype introgression’ (Bevan et al., 2017). Nevertheless, the lack of information regarding the superior haplotype combinations of several key grain quality genes has been one of the major bottlenecks, and the 3000-rice genome project (3K-RGP) offers enormous potential for harnessing the haplotype diversity of grain quality genes in rice (Li et al., 2014b).

Genome-wide association studies (GWAS) have become popular for the genetic dissection of complex traits into QTL/candidate genes that might be deployed in precision breeding programs aimed at crop improvement (Lipka et al., 2015, Tibbs et al., 2021). It is considered more efficient than bi-parental mapping approaches considering the naturally occurring genetic diversity, high-density genetic markers, and fewer linkage disequilibrium to identify candidate genes (Alqudah et al., 2020). Statistical methods with varying degrees of reliability substantially influence the significant MTAs determined by GWAS (Gawenda et al., 2015; Visscher et al., 2017; Wen et al., 2018). The commonly used single-locus mixed model independently scans each SNP marker for association with a phenotypic trait (Waugh et al., 2014; Gupta et al., 2019). However, this model lacks accuracy in estimating the SNP effects and identifies false negatives if the desired trait is governed by many genes at different loci (Wang et al., 2016), which is a common scenario in most quantitative traits or in case it requires a Bonferroni correction (Wen et al., 2018). It has also been proposed that single-locus models fail to detect the epistatic interactions that may exist between the closely linked genes (Gawenda et al., 2015) and are less suitable for harnessing the haplotype diversity of genes of interest that exist in the germplasm (Lu et al., 2011; Contreras-Soto et al., 2017; N’Diaye et al., 2017). To overcome the shortcomings of single-locus models, multi-locus models such as multi-locus random SNP-effect MLM (mrMLM) (Wang et al., 2016); multi-locus mixed model (MLMM) (Segura et al., 2012), iteractive modified sure-independence screening expectation maximization Baysian least absolute shrinkage and selection operator (ISIS EM-BLASSO) (Tamba et al., 2017), FASTmrMLM (multi-locus random SNP-effect) (Tamba and Zhang, 2018), FASTmrEMMA (fast multi-locus random-SNP-effect efficient mixed model analysis) (Wang et al., 2016), polygenic-background-control-based least angle empirical Bayes (pLARmEB) (Zhang et al., 2017), and integration of Kruskal–Wallis test with empirical Bayes (pKWmEB) (Ren et al., 2018) were developed that test multiple SNP markers simultaneously to capture the molecular basis underlying different complex traits in different crop species (Wang et al., 2016) by overcoming the strong population structure and high linkage disequilibrium between the markers. In this investigation, we performed a GWAS and conducted a candidate gene-based association study in a set of 3K-RGP panels, analyzed the haplotype diversity of candidate genes, and evaluated the performance of different haplotypes associated with grain aroma, head rice recovery (HRR, %), and percentage of grains with chalkiness (PGC, %) to accelerate the design of next-generation quality-rich rice varieties by incorporating superior haplotypes for use in future rice improvement programs.

## Materials and methods

### Plant materials and phenotyping

A subset panel of 3K re-sequenced genomes (https://doi.org/10.1186/2047-217X-3-7) was obtained from the IRRI South Asia Regional Center, NSRTC Campus, Varanasi, Uttar Pradesh, India. The 196 rice accessions used in our investigation were collected from 89 countries belonging to four major populations: *Xian(indica)* (171), *aus/boro* (22), *tropical Geng (japonica)* (3), intermediate type (2), and two semi-dwarf varieties Pusa Basmati 1121 and PB-1 (**Supplementary Table 1**). The 198 accessions were planted in randomized plots in the field at the ICAR-Indian Agricultural Research Institute (IARI), New Delhi, India with four replications within Kharif 2020 and Kharif 2021. The uniform growth of seedlings was confirmed by germinating seeds on a raised seedbed, and 21 days old plantlets were transplanted. Each accession was sown in two rows, with each row consisting of 10 plants at a distance of 20 cm × 15 cm within and between the two rows. Standard practices were followed for field management. At maturity, paddy seeds from each plot were collected in bulk and dried in hot air ovens. Approximately 150 g of seeds was dehusked and milled in a laboratory rice husker and milling machine (model JGMJ 8098, China) after cleaning the paddy with the optimal level of moisture. Three traits related to grain quality were recorded using the Standard Evaluation System in rice (http://www.knowledgebank.irri.org/images/docs/rice-standard-evaluation-system.pdf): grain aroma, head rice recovery (HRR, %), and percentage of grains with chalkiness (PGC, %). The grain aroma was estimated for each accession using a sensory method (Sood and Siddiq, 1978). Two fragrant Basmati rice varieties, *viz.*, Pusa-1121 with an aroma score of 3, PB-1 with an aroma score of 2, and a non-aromatic rice Pusa-44, were used in the analysis, and each sample was evaluated by seven experts to confirm the phenotype. Following milling, head rice recovery (HRR, %) and percentage of grains with chalkiness (PGC, %) were calculated manually and using a stereomicroscope based on the SES Scale 9, respectively. Meanwhile, the range, mean value, deviation, and phenotypic coefficient of variation (CV) were calculated for each trait using R Studio (**Supplementary Table 2**). Correlations of quality traits among themselves were also studied by measuring the linear correlation calculated using the R package corrr (https://cran.r-project.org/bin/windows/base/). Heritability was estimated for all three quality traits using R package variability.

### Genotyping

The genomic data of 198 accessions selected from the 3K RG panel were analyzed. The SNP dataset (3K RG 1M GWAS SNP) was downloaded from the repository of rice variants in the public domain SNP-seek(http://snp-seek.irri.org/_download.zul). Missing data were imputed using Beaglev5.4 software. Quality control was performed using TASSELv5.2.82 software to obtain a filtered subset of 553,831 SNPs with a minor allele frequency >5% and a major allele frequency <95% for genome-wide association analysis.

### Cluster analysis, population structure, and kinship

Neighbor-joining clustering was performed based on the SNP data using TASSELv5.2.82 software and visualized using the interactive tree of life (iTOL) software. The subgroups were assessed using a Bayesian model-based approach in STRUCTUREv2.3.4 (Pritchard et al., 2000) and PCA analysis. The structural analysis was executed with the presumed number of subgroups ranging between one and seven, with each K repeated thrice. A burin-in period of 100,000 iterations followed by 100,000 Markov Chain Monte Carlo (MCMC) simulations were implemented for every run, and the number of subgroups was then determined using the Evanno ΔK method (Evanno et al., 2005) embedded in the STRUCTURE HARVERSTER software (Earl and VonHoldt, 2012). Component analysis was performed using the Genome Association and Prediction Integrated Tool (GAPIT) R package (Lipka et al., 2012). Number of significant principal components explaining the population variance and structure were determined by plotting a scree plot in R. For kinship calculation, the Centered_identity-by-state (IBS) default method was employed in TASSELv5.2.82 software (Bradbury et al., 2007). The structure, kinship matrix, and average trait value of each accession were used for the association studies based on SNP data.

### Linkage disequilibrium analysis

Linkage disequilibrium (LD) decay distance between the pair of SNP markers was calculated on each chromosome as the squared coefficient of correlation (r^2^) values of alleles using LDkit software. The position on the chromosome at which the r^2^ value reduced to half of its average maximum value was defined as the decay in LD (Huang et al., 2010).

### Candidate gene-based association analysis and identification of superior haplotypes

We performed GWAS on 198 rice accessions using the MLM and CMLM model with filtered 553,831 SNP markers and default settings in GAPIT software to estimate the significant SNP-MTAs for grain aroma, HRR%, and PGC%. Three multi-locus models, namely mrMLM, FASTmrMLM, and FASTmrEMMA, were also constructed using the mrMLM R package (https://cran.r-project.org/web/packages/mrMLM/index.html) to accurately detect the candidate QTN effect values and confirm the true associations. Considering an LOD score value ≥3 as the threshold, significant QTNs were identified (Duan et al., 2017). The common QTNs detected by the two different ML-GWAS models and SL-GWAS models were predicted to be good candidates for rice quality traits. Local haplotype blocks of each robust QTN were generated with all filtered SNP using PLINKv1.9 (www.cog-genomics.org/plink/1.9/) as per standard methodology (Gabriel et al., 2002). LD heatmaps were generated using the LDBlockShow tool. All genes located within the LD decay distance of the identified QTNs were extracted and subjected to comprehensive gene annotation studies to identify the candidate loci for each quality trait using The Rice Annotation Project-Database (RGAP, http://rice.uga.edu/), Information Commons for Rice (IC4R, http://ic4r.org/), and Gramene (https://www.gramene.org/) databases and used for gene mining. The haplotypes for each of these candidate loci were estimated considering the non-synonymous coding SNPs in the SNP-Seek database (https://snp-seek.irri.org/), and Student’s t-test was performed to test the significant differences among the haplotypes. The haplotypes revealed and the phenotypic distribution of each grain quality trait were then represented as boxplots using the ggplot2 package in R Studio.

## Results

### Trait variance and correlations

**Figure 1:**
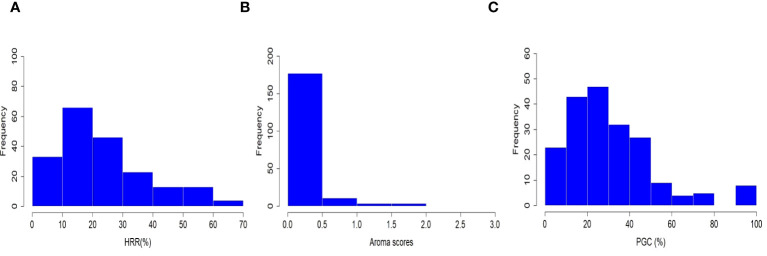
Phenotypic distribution of head rice recovery (HRR, %), grain aroma (AR), and percentage of grains with chalkiness (PGC, %) in a subset of 198 rice accessions.

Three grain quality-related traits, grain aroma, head rice recovery (HRR, %), and percentage of grains with chalkiness (PGC, %), were investigated in the selected subset of 198 accessions sampled from 3,000 re-sequenced genomes in the IRRI Rice Genome Project (3K-RGP). Rice accessions consisting of a diverse set of *Xian*, *japonica*, *aus/boro*, intermediate type cultivars, and two check varieties *viz.*, PB-1121 and PB-1 were planted at the research field of ICAR-IARI, New Delhi in 2020 and 2021. The statistical parameters were estimated, and the results are listed in **Supplementary Table 2**. HRR (%) and PGC (%) followed a negatively skewed distribution, whereas the grain aroma followed a positively skewed distribution (**Figure 1**). Furthermore, correlation analysis among the three traits indicated a statistically significant variation between the paired quality traits at the 5% and 1% levels of significance, except for the relationship between HRR (%) and PGC (%). Grain aroma was positively associated with HRR (%) (PCC = 0.28) and negatively associated with PGC (%) (PCC = −0.17), which is consistent with several previous studies (Sanchez et al., 2023; Song et al., 2007; Adjah et al., 2020; Qiu et al., 2021). In addition, HRR (%) and PGC (%) had a very weak positive correlation with a Pearson correlation coefficient (PCC) of 0.03, which was also consistent with current correlation studies and BLUP estimates (Sanchez et al., 2023; Nirmaladevi et al., 2015; Vemireddy et al., 2015; Cruz et al., 2021; Ali et al., 2023). Broad-sense heritability (H^2^) estimates were high for HRR (%) (0.99) and PGC (%) (0.98) which was consistent with similar studies (Sanchez et al., 2023; Ali et al., 2023). The considerably low H^2^ for grain aroma (0.28) suggested that its environmental influence was attributed to the experimental conditions, as pointed out in an earlier study (Vemireddy et al., 2015). These findings indicate a close relationship among the abovementioned quality traits and suggest their potential role in the genetic improvement of rice grain yield.

### Population structure and linkage disequilibrium analysis

**Figure 2:**
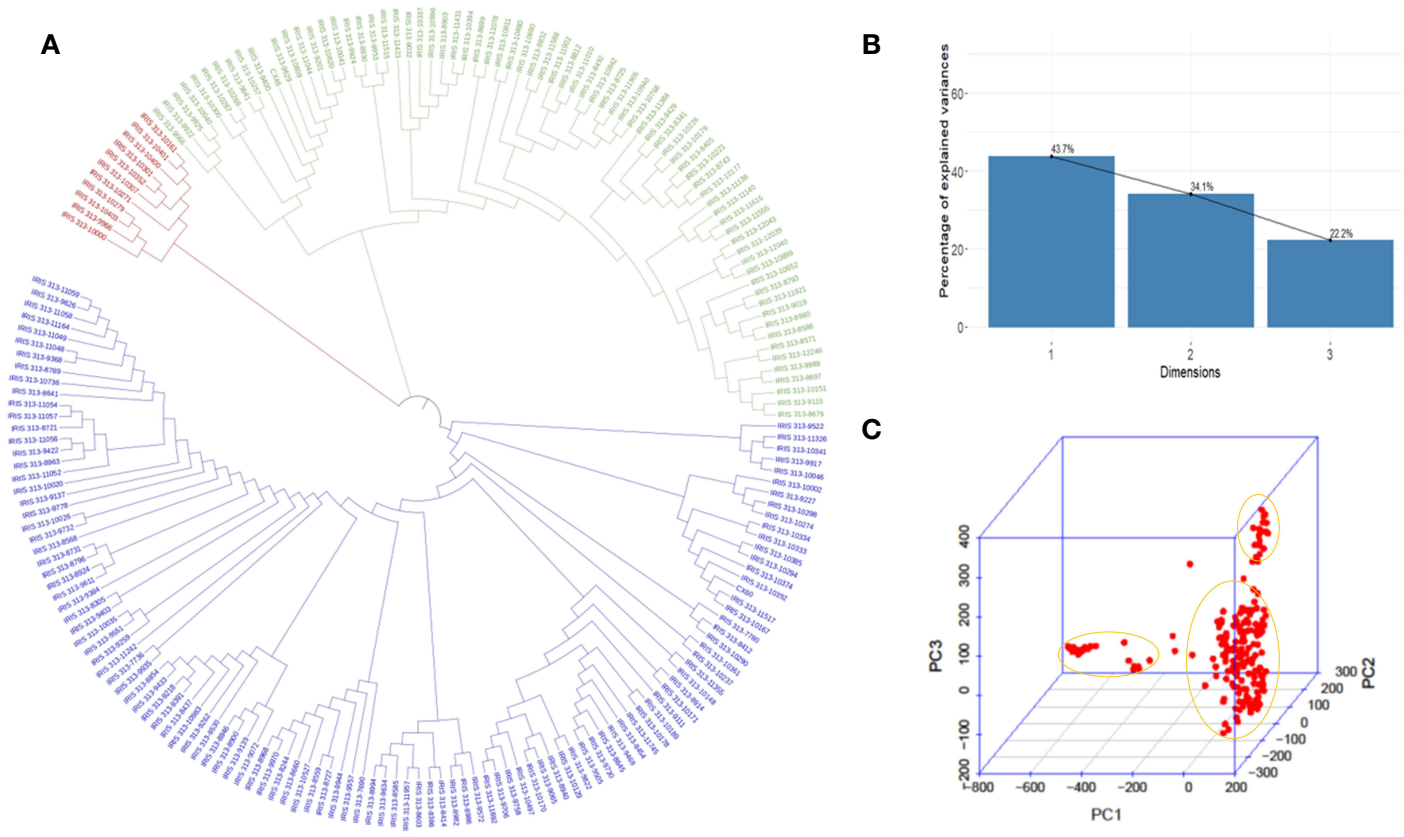
Model and PCA based analysis of genetic structure of 198 rice accessions. **(A)** NJ clustering of 198 rice accessions constructed using 5,53,229 SNPs evenly distributed throughout the genome. **(B)** Scree plot depicting the genetic variation with principal components. PC1, PC2, and PC3 represent the first, second, and third principal components, respectively. **(C)** Biplot depicting three clusters identified in the selected rice panel.

**Figure 3:**
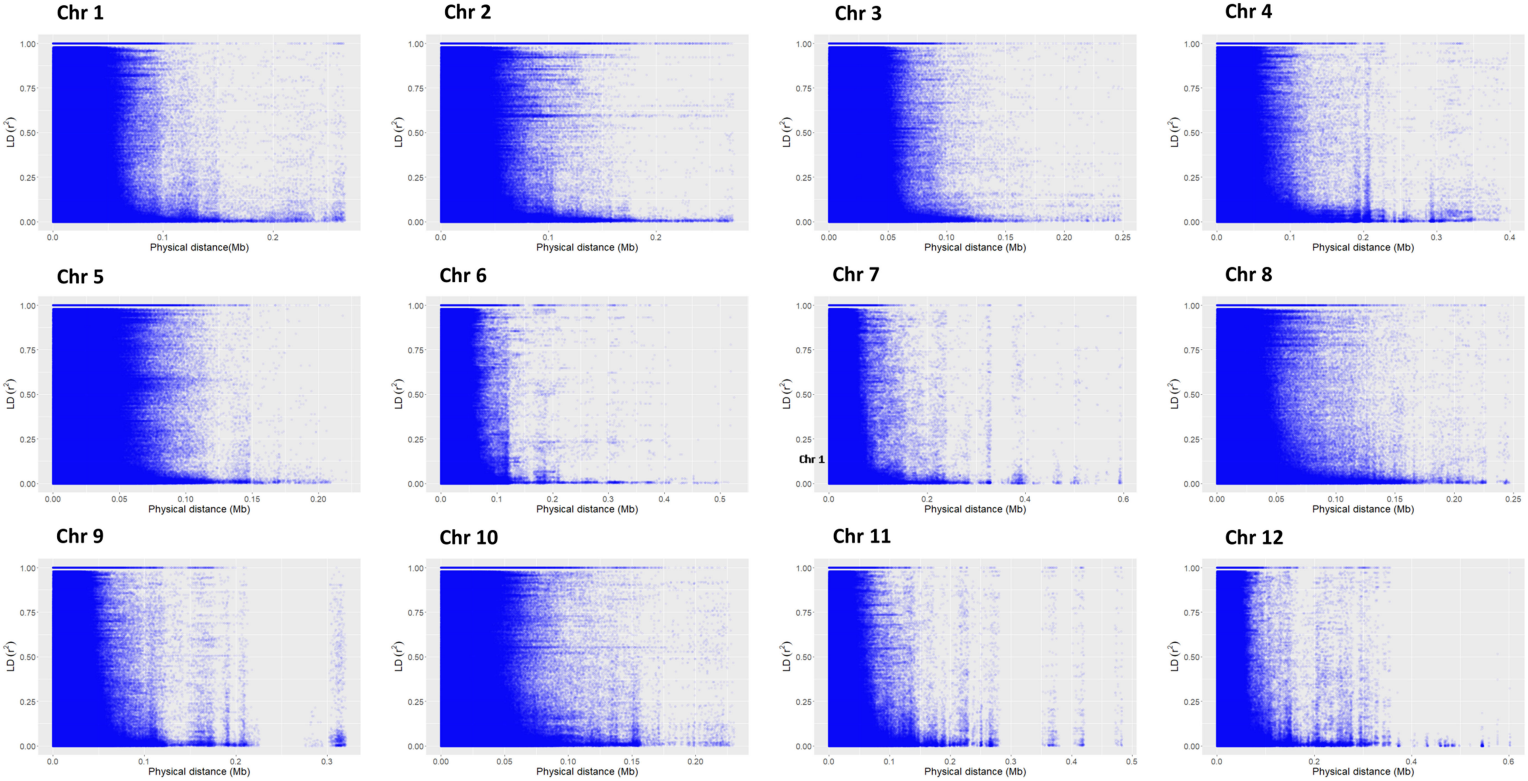
Chromosome-wise linkage disequilibrium decay based on 198 accessions. The decline in LD-r^2^ between SNP markers is presented as a function of physical distance in base pairs.

According to principal component analysis, there were three subpopulations in the selected rice panel (**Figure 2C**). The scree plot suggested the significance of three PCs in the subset selected, with the first two PCs (PC1 and PC2) explaining a cumulative percent variance of 77.8 (**Figure 2B**). Neighbor-joining (NJ) clustering also revealed three distinct clusters based on genetic distances derived from SNP differences in the selected rice accessions (**Figure 2A**). Cluster 1 was identified as the smallest cluster consisting of 4.04% of *indica* rice accessions belonging to *indx* and *ind1b* subpopulations. A total of 26.26% of the *Xian* subpopulations, *viz., ind1a*, *ind1b*, *ind2*, and *ind3*, were included in cluster 2. However, cluster 3 was recognized as the largest and the most diverse cluster, comprising 69.69% of the total accessions, were *Xian*, *japonica*, *aus/boro*, and *intermediate*-type subpopulations. LD decay analysis was conducted using the filtered SNPs. Maximum r^2^ estimated on the 90th percentile of chromosomes 1 to 12 was 0.3, 0.25, 0.35, 0.25, 0.35, 0.3, 0.3, 0.25, 0.3, 0.35, 0.25, and 0.25, respectively. As shown in **Figure 3**, variations were observed in the LD decay distance among the 12 chromosomes, with the fastest decay occurring in chromosome 12. These SNPs were found to be distributed across the whole rice genome, with an average number of SNP per kb 1.28 sufficiently dense to identify significant associations and QTLs.

### Association analysis

**Figure 4:**
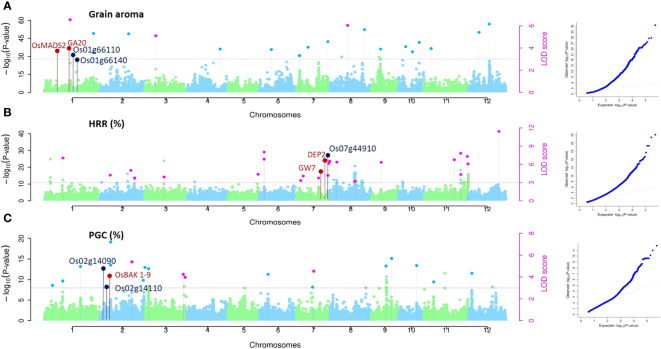
GWAS for grain quality traits in rice accessions. Manhattan and Quantile-Quantile plots derived through the mrMLM, FASTmrMLM, and FASTmeEMMA methods depicting the distribution of QTNs on 12 rice chromosomes for grain aroma (AR), head rice recovery HRR (%), and percentage with grain chalkiness PGC (%). Pink dots indicate all the QTNs mapped by more than one GWAS method, while all the QTNs identified by a single method are indicated by the light colored dots shown above the gray dotted lines. The known genes around QTNs are marked in red, and putative candidates around the identified QTNs are marked in dark blue.

Associations for all three traits (Aroma, HRR (%), and PGC (%)) were studied using single-locus approaches (MLM and CMLM) for QTL detection and three multi-locus methodologies (mrMLM, FASTmrMLM, and FASTmrEMMA) to identify QTNs. Using the MLM method, 198, 198, and 198 single nucleotide polymorphic (SNP) markers corresponding to 23, 22, and 32 QTLs were found to be associated with aroma, HRR (%), and PGC (%), respectively, considering the threshold value of -log (P) value = 3 (**Supplementary Table 3**), similar to multiple recent GWAS studies (Kikuchi et al., 2017; Bheeanahalli et al., 2021; Hu et al., 2022). Of these, 24 QTNs for aroma using mrMLM (10), FASTmrMLM (11), and FASTmrEMMA (3). For HRR (%), eight, 11, and four QTNs were detected using mrMLM, FASTmrMLM, and FASTmrEMMA, respectively, and 23 QTNs were correlated with PGC (%) using mrMLM and FASTmrMLM (**Supplementary Figure 1**). Manhattan and quantile-quantile plots of all the three quality traits presented in **Figure 4** implied that false associations were controlled and the SNPs detected by ML-WAS methods were true associations; however, we witnessed inflation in Q–Q plots with incorporated population structure. This inflation persisted because the mixed linear approach (accounting for structure) utilized the first three PCs as covariates in the regression. However, the PC-adjusted model-based estimates of standard errors remove the structure problem, providing correctly calibrated p-values, which has been well documented in several studies (Price et al., 2006; Zhang et al., 2008; Voorman et al., 2011). One of the QTNs detected for aroma (qAR-1-1) was located in proximity to the well-known rice fragrance gene *Badh2* (151 kb). The recessive gene *BADH2* is well established to govern the synthesis of 2-acetyl-1-pyroline (2-AP) in aromatic rice (Imran et al., 2022). Furthermore, we found that *qHRR-3-1* existed in the same region adjacent to *OsRLCK113* (cysteine-rich receptor-like kinase 28 precursor gene, *LOC_Os03g31260*) (Li et al., 2022) and the gene encoding the ring zinc finger protein (*LOC_Os03g31320*) (65–66), with confirmed roles in controlling grain yield and quality traits in rice*. qPGC-3-1* and *qPGC-3-2* were located adjacent to *OsLTP1.3* (Ltpl28-Seed Storage/Protease Inhibitor/Ltp Family Protein Precursor, *LOC_03g59380*), *OsCESA2* (Cellulose Synthase, *LOC_03g59340*) and *OsCPK8* (Camk_Camk_Like.24- Calcium Dependent Protein Kinases, *LOC_03g59390*) genes regulating grain quality traits in rice. Similarly, *qPGC-7-2* overlapped with a gene encoding a retrotransposon protein located in the vicinity of no apical meristem genes *ONAC65* (*LOC_07g27330*) and *ONAC102* (*LOC_07g27340*), which serves as a regulator of starch and accumulation of proteins, thereby improving grain quality in rice (Wang et al., 2020).

**Figure 5:**
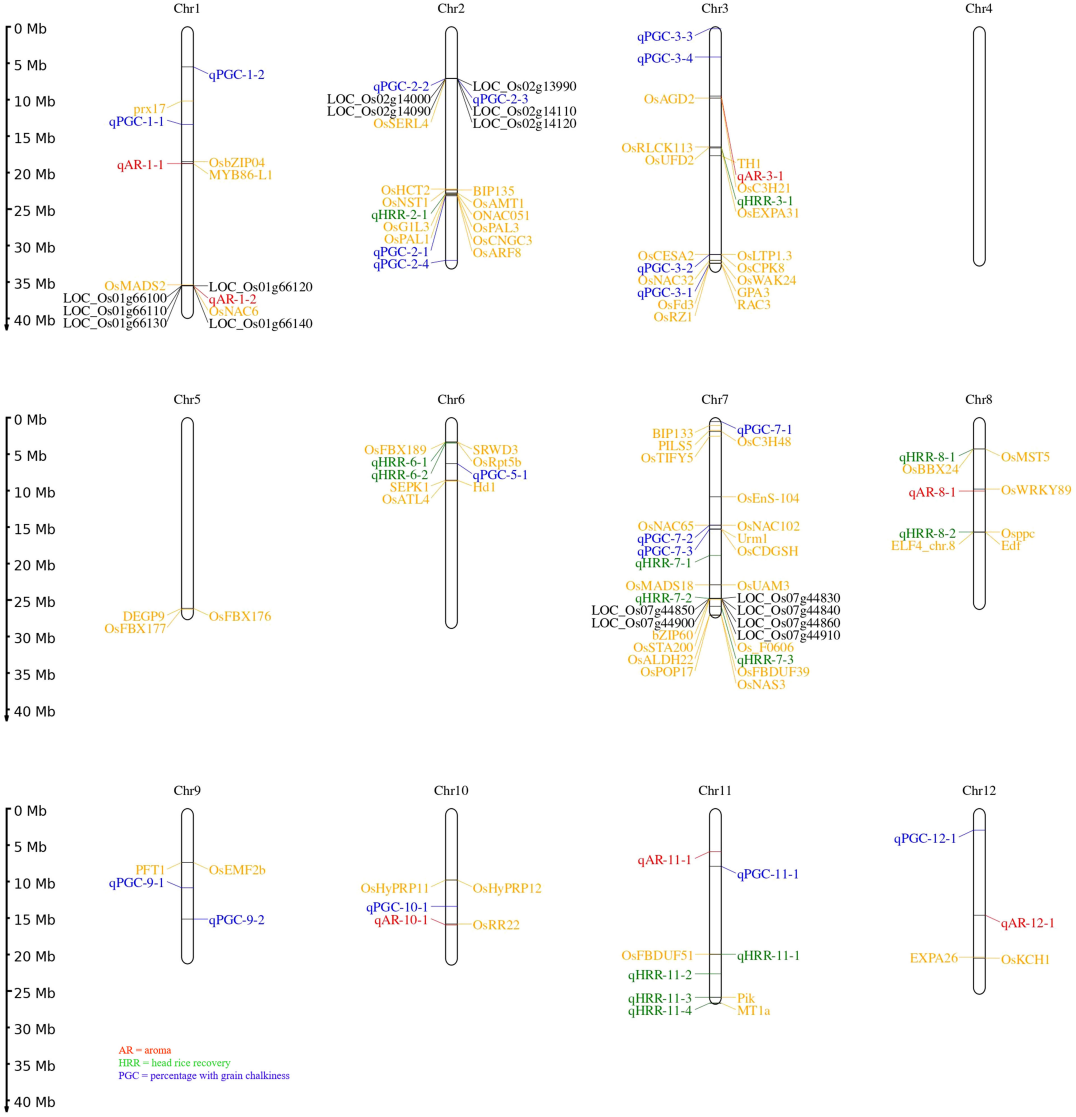
Chromosomal distribution of all loci for grain quality traits using MLM, CMLM, mrMLM, FASTmrMLM, and FASTmeEMMA. The naming of QTNs starts with a letter ‘q’ subsequently followed by two or three letter identifiers and the chromosome number. In case numerous QTNs are mapped for a quality trait on corresponding chromosome at that point naming is done based on their relative location on the chromosome. Seventy-five known genes are labelled with yellow script; black color represents candidate genes for the quality traits under study.

In addition, assessment of the results of SL-GWAS and ML-GWAS revealed 39 QTNs in common based on a critical LOD score ≥3, explaining 0.03%–9.57% of the phenotypic variation (R^2^) (**Table 1**). The fact that half of the detected QTNs (19/39) overlapped with previously reported genes/QTLs supports the consistency of our results. Among these, 7, 13, and 19 were associated with AR, HRR (%), and PGC (%), respectively. Seven candidate QTNs significantly related to AR were located on chromosomes 1, 3, 8, 10, 11, and 12. For HRR (%), 13 putative QTNs were found to be distributed on chromosomes 2, 3, 6, 7, 8, and 11. A total of 19 QTNs correlated with PGC (%) were found to be located on chromosomes 1, 2, 3, 5, 7, 9, 10, 11, and 12. Of these, four QTNs were detected by both SL-GWAS and at least two ML-GWAS methods (qPGC-2-1, qPGC-3-1, qPGC-3-2, and qPGC-7-3). As many as 75 cloned genes were closely associated with rice yield and appearance quality within the genomic ranges ( ± 100 kb) of the 39 QTNs detected by the SL-GWAS and ML-GWAS methods (**Figure 5**; **Supplementary Table 4**).

**Table 1:** QTNs for the three quality traits detected concurrently by using single- and multi-locus GWAS methodologies.

Trait	QTN	Chr	Position	LOD	R^2^(%)^1^	Method^2^	LOC^3^/QTL^4^
*Aroma (AR)*	qAR-1-1	1	20227999	0.05–9.03	0.05–7.5	1,2,3,4,5	
	qAR-1-2	1	38383904	3.59	3.16	1,2,3	GA20ox-2
	qAR-3-1	3	10338993	4.46–5.74	0.13–5.89	1,3,4	
	qAR-8-1	8	10892476	5.54–6.54	4.25–4.93	3,4	LTP48/CQAP1
	qAR-10-1	10	17265187	4.503	0.09–2.57	1,2,5	OsCESA7
	qAR-11-1	11	6394202	3.9514	0.03–6.53	1,2,3	OsSRP-PLP
	qAR-12-1	12	15819670	6.1642	0.09–3.94	1,2,5	CQAP3
*Head Rice Recovery* *HRR (%)*	qHRR-2-1	2	24752396	5.0096	0.05–5.71	1,2,5	hwh1, AQCV031^a^
	qHRR-3-1	3	17840988	3.9148	0.09–4.47	1,2,4	LOC_Os03g31310
	qHRR-6-1	6	3667482	6.8549	0.08–8.20	1,2,3	
	qHRR-6-2	6	3730045	8.0183	0.09–5.51	1,2,3	
	qHRR-7-1	7	20413747	3.7534	0.05–3.97	1,2,5	LOC_Os07g34130
	qHRR-7-2	7	26771672	4.1753	0.06–4.47	1,2,5	LOC_Os07g44830
	qHRR-7-3	7	28019959	6.168	0.06–6.95	1,2,3	
	qHRR-8-1	8	4580996	6.3682	0.05–7.59	1,2,5	
	qHRR-8-2	8	16979079	3.2005	0.07–2.00	1,2,4	
	qHRR-11-1	11	21623134	6.7537	0.06–4.22	1,2,4	LOC_Os11g36640
	qHRR-11-2	11	24456311	7.8223	0.06–7.02	1,2,4	
	qHRR-11-3	11	27996997	7.3025	0.07–4.78	1,2,4	OsPCBP
	qHRR-11-4	11	28857401	6.0719	0.05–5.69	1,2,3	OsRhmbd18
*Percentage with grain chalkiness PGC (%)*	qPGC-1-1	1	14474816	3.6322	3.59	1,2,3	LOC_Os01g25530
	qPGC-1-2	1	5928150	3.2289	5.51	1,2,3	LOC_Os01g11110
	qPGC-2-1	2	25081182	3.54–7.22	8.45–9.57	1,2,3,4	LOC_Os02g41720
	qPGC-2-2	2	7633393	7.19	6.15	1,2,3	LOC_Os02g13990, AQGB108^b^
	qPGC-2-3	2	7660595	5.08	2.95	1,2,4	AQGB109^c^, AQGB084^d^
	qPGC-2-4	2	34652183	3.69	1.55	1,2,4	LOC_Os02g56565
	qPGC-3-1	3	35098972	3.75–4.18	2.08–4.98	1,2,3,4	
	qPGC-3-2	3	33802678	3.99–4.48	2.8-6.1	1,2,3,4	
	qPGC-3-3	3	326950	4.87	3.52	1,2,3	
	qPGC-3-4	3	4530119	4.76	3.34	1,2,4	
	qPGC-5-1	5	6812458	4.23	8.51	1,2,3	
	qPGC-7-1	7	620874	6.4849	0.07–1.14	1,2,3	
	qPGC-7-2	7	15975911	3.07	2.35	1,2,4	LOC_Os07g27420
	qPGC-7-3	7	16539429	3.9–5.16	3.9–5.37	1,2,3,4	
	qPGC-9-1	9	11755843	5.0082	2.7252	1,2,4	
	qPGC-9-2	9	16443727	5.696	3.528	1,2,4	
	qPGC-10-1	10	14524700	5.05	3.21	1,2,4	
	qPGC-11-1	11	8517398	3.5495	2.8693	1,2,4	
	qPGC-12-1	12	3125286	4.3352	3.4133	1,2,4	

^1^R^2^(%): phenotypic variance explained.

^2^Methods 1–5 represent MLM, CMLM, mrMLM, FASTmrMLM, and FASTmrEMMA, respectively.

^3^Locus name based on MSU 7.0.

^4^QTL ID based on Gramene QTL Database. ^a^ Li et al. (2003); ^b,c,d^ Wan et al. (2005).

### Mining of potential candidate loci

We selected common QTNs mapped using the SL-GWAS and ML-GWAS algorithms for a detailed study. The candidate genes were identified based on haplotype analysis of non-synonymous coding SNPs in each candidate gene located inside the LD block defined for the selected QTN.

**Figure 6:**
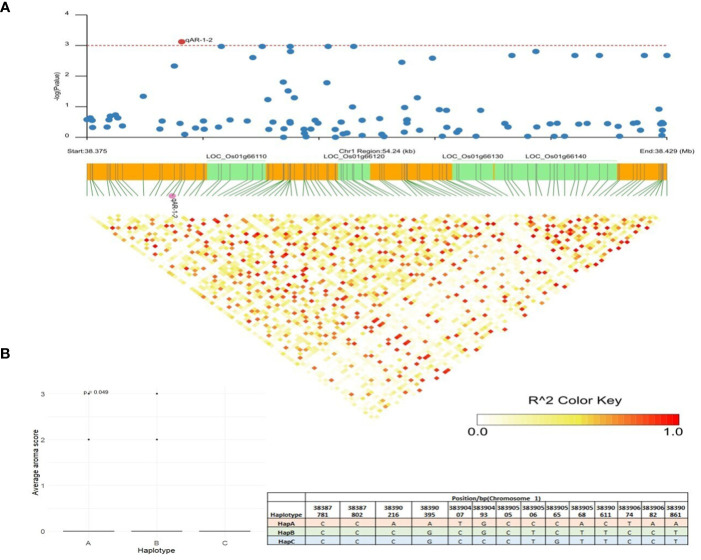
Haplotype analysis of LOC_Os01g66110. **(A)** Linkage disequilibrium (LD) based heatmap for qAR-1-2. **(B)** Boxplot for grain aroma depicting three allelic combinations of LOC_Os01g66110. X-axis shows the three different alleles of LOC_Os01g66110 and Y-axis shows the average aroma scores.

qAR-1-2, located at 38,383,904bp on chromosome 1, showed association signals with grain aroma using MLM, CMLM, and mrMLM methods with a Logarithm of Odds (LOD) score of 3.59% (**Table 1**). A total of 54 kb LD block (38,375,000 bp–38,429,000 bp) was generated (**Figure 6A**) as per the method described above (Gabriel et al., 2002). Gene annotations suggested five candidates for this block: *LOC_Os01g66100* (gibberellin20oxidase2 gene, *OsGA20ox2*), *LOC_Os01g66110* (a methyltransferase), *LOC_Os01g66120* (no apical meristem protein-encoding gene, *OsNAC6*), *LOC_Os01g66130* (an armadillo/beta-catenin repeat family protein, *OsPUB16*), and *LOC_Os01g66140* (plus-3 domain-containing protein). Among these, *LOC_Os01g66110* is the most likely gene because the heavy metal transporter genes involved in the biosynthesis of 2-AP, which determines the aroma in fragrant rice, are known to be regulated by DNA methylases via active histone modifications (Imran et al., 2022). Missense mutations in *LOC_Os01g66110* resulted in three allelic combinations. Genotypes with superior HapA exhibited higher average aroma scores, whereas genotypes with HapB and HapC showed lower aroma scores (**Figure 6B**). Another candidate gene, *LOC_Os01g66140*, directly interacts with histone H4 and zinc ions, explaining its role in 2-AP biosynthesis. Three haplotypes were observed for *LOC_Os01g66140*, and haplotype A showed a significantly higher average aroma score than the other two haplotypes.

**Figure 7:**
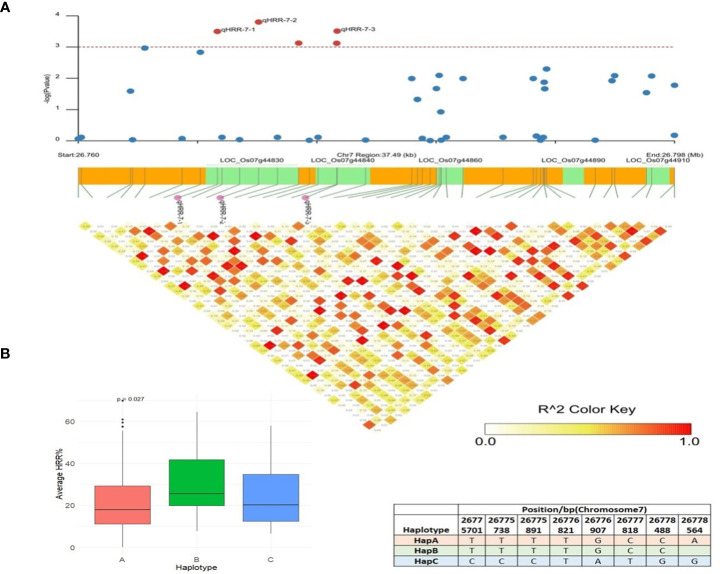
Haplotype analysis of LOC_Os07g44910. **(A)** Linkage disequilibrium (LD) based heatmap for qHRR-7-2. **(B)** Boxplot of HRR (%) trait depicting three allelic combinations of LOC_Os07g44910. The X-axis shows three different alleles of LOC_Os07g44910 and the Y-axis shows average HRR (%).

The SL-GWAS and ML-GWAS test results verified peaks on chromosome 7 for HRR (%). qHRR-7-2, located at 26,771,672 bp and encoding a proline-rich family protein, was significantly linked to HRR (%) with the FASTmrEMMA method with an LOD score of 4.17 (**Table 1**). Using MLM, this SNP showed associations with HRR (%) with a high level of significance (p = 3.84 × 10^−6^) and an R^2^ of 5.43%. An LD block of 26,760,000 bp to 26,798,000 bp was constructed using pairwise estimation of LD (**Figure 7A**). The fine mapping of this genetic region associated with HRR (%) identified five candidate genes using genome annotation tools: *LOC_Os07g44830* belonging to the proline-rich family, *LOC_Os07g44840* encoding a transposon with unknown function, and *LOC_Os07g44850*, *LOC_Os07g44860*, *LOC_Os07g44900*, and *LOC_Os07g44910* are gibberellin receptor protein-encoding genes. The *LOC_Os07g44910*, annotated as putatively expressed gibberellin receptor GID1L2 protein, showed significant differences in HRR (%) between the haplotypes (**Figure 7B**). Therefore, HapA is a superior genotype and rice accessions with a higher frequency of HapA could be selected from the current panel to improve head rice recovery (%) in rice. Earlier studies clearly indicated the role of Gibberellic Acid in controlling panicle architecture and yield traits in rice (Deveshwar et al., 2020). Moreover, *LOC_Os07g44910* colocalized with the *dense and erect panicle 2* (*DEP2*) gene, which is mainly involved in rachis elongation and branching in panicles (Li et al., 2003; Wan et al., 2005), and the *GW7* gene, which encodes a TONNEAU1-recruiting motif protein that improves grain yield and quality by directly interacting with *GW8* (*OsSPL16*) (Li et al., 2010; Reig-Valiente et al., 2018). We utilized the IC4R database to confirm the functional role and analyzed the expression profile data of *LOC_Os07g44910* in rice and found that the gene encodes an alpha/beta hydrolase fold-3 domain-containing protein with the highest expression in the seedlings and young shoots. Previous studies have reported that the *D14* gene encoding an alpha/beta hydrolase family protein inhibits rice tillering via the strigolactone signaling pathway (Gao et al., 2009; Wang et al., 2015; Guo et al., 2020); thus, it is likely that *LOC_Os07g44910* influences grain yield in rice.

**Figure 8:**
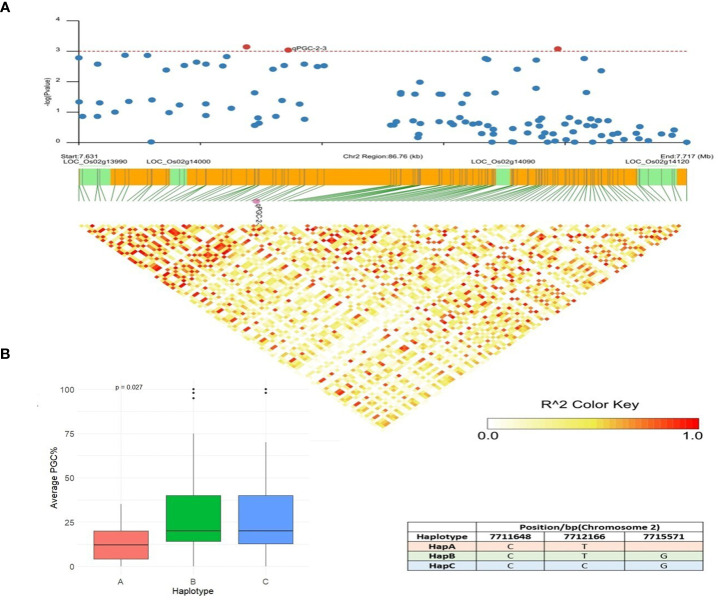
Haplotype analysis of LOC_Os02g14120. **(A)** Linkage disequilibrium (LD) based heatmap for qPGC-2-3. **(B)** Boxplot of PGC (%) trait depicting three allelic combinations of LOC_Os02g14120. X-axis shows three different alleles of LOC_Os02g14120 and Y-axis shows average PGC (%).

*qPGC-2-3* was another QTN detected by multiple models and showed associations with the percentage of grains using chalkiness FASTmrMLM methods with an LOD value of 5.08. This QTN was also detected by the MLM and CMLM methods with a p value of 3.05 × 10^−6^. An LD block was defined for this QTN (83.63 kb), and five candidate genes were identified in this region (**Figure 8A**). *LOC_Os02g13990* (U2 small nuclear ribonucleoprotein A) and *LOC_Os02g14000* (actin-related protein 2/3 complex subunit 3) only had synonymous SNPs with a -log (P) value less than 3. *LOC_Os02g14120* is a Brassinosteriod Insensitive 1 Associated Receptor Kinase 1 precursor gene (*OsBAK 1-9*). Non-synonymous mutations in *OsBAK 1-9* resulted in three major haplotypes: HapA, HapB, and HapC. The accessions with favorable HapA displayed lower PGC (%) than accessions with HapB and HapC types (**Figure 8B**). The identified favorable allele and functional site in *LOC_Os02g14120* reduces the degree of chalkiness in rice by breeding. Differences in rice grain quality have been attributed to the regulation by a set of other genes involved in multiple pathways that influence grain appearance quality. *LOC_Os02g14110* is annotated as an aminotransferase, Class I and Class II domain-containing protein gene, and the third candidate gene, *LOC_Os02g14090*, is a berberine and berberine-like domain-containing protein gene. Previous research has also verified that brassinosteroid-associated receptor kinase genes, putatively expressed aminotransferases, and berberine and berberine domain-containing protein genes govern quality traits, *viz.*, chalkiness and grain shape (Biselli et al., 2015) in rice, which led us to hypothesize that *LOC_Os02g14120*, *LOC_Os02g14110*, and *LOC_Os02g14090* may be rice grain PGC (%) regulatory genes.

## Discussion

Increasing living standards underline the need to develop healthier high-quality rice (Yu et al., 2013; Hori et al., 2015; Sahu et al., 2017; Wang et al., 2017; Misra et al., 2019; Meng et al., 2022) for traits such as color, aroma, lack of broken seed grains, grain length, and flavor. To meet consumer preferences and market demands, the development of tailored rice with preferred appearance quality is of utmost importance after rice yield enhancement (Arite et al., 2009; Abbai et al., 2019; Selvaraj et al., 2021). Grain quality is a complex quantitative trait (Yu et al., 2013; Hori et al., 2015; Misra et al., 2019; Meng et al., 2022) governed by manifold genes, and there is a large gap in our perception of the networks regulating grain quality in rice (Li et al., 2022). GWAS has become a robust tool for the rapid identification of genetic factors (Adjah et al., 2020) associated with traits governed by several genes in crop plants that are diverse and provides goals for future efforts aimed at rice improvement (Zhou et al., 2020). However, breeding by design has achieved limited success because of the lack of information on the correct genetic loci of desired traits and precision in deciphering the favorable haplotype combinations of these genes dissected to date (Fitzgerald et al., 2009; Abbai et al., 2019; Selvaraj et al., 2021).

Resequencing-based germplasm lines enable the detection of pre-existing variations, functional sites of genes, and novel alleles associated with traits of interest (Begum et al., 2015), which may be explored by GWAS analysis. In this context, the abundant genetic variations in 3K RG resequencing projects make it a valuable reservoir of gene diversity and a prospective source of elite genes that can be deployed in rice breeding (Abbai et al., 2019; Selvaraj et al., 2021). Traditional single-locus models, which are commonly adopted to identify genetic variants in several cereal crops, have some limitations, neglecting small-effect QTLs in particular. Lower false positives and higher statistical predictions of multi-locus algorithms have been established by many association studies (Yuan et al., 2017; Zhang P. et al., 2019), and researchers usually combine facts about different ML-GWAS models to mine the genes that control complex traits.

In the present study, we adopted two SL-GWAS methods and three ML-GWAS methods to assess three quality traits of 198 selected rice accessions (a subset of 3K RGP). Subsequently, 198,198 and 198 significant SNPs, while 23,22 and 32 QTLs were identified by MLM underlying AR, HRR (%), and PGC (%), respectively (**Supplementary Table 3**). Similarly, 24,23 and 23 significant QTNs were detected using ML-GWAS methodologies associated with the abovementioned three quality traits (**Supplementary Figure 1**). Interestingly, the QTNs mapped by multi-locus GWAS analysis were more dispersed than those mapped by the MLM and CMLM methods. The significant loci detected by the MLM method, for example, were confined to specific chromosomes, indicating its failure to identify new loci across the entire rice genome. Several QTNs identified by multi-locus methods were distributed across the other chromosomes, among which 39 common QTNs were considered powerful, robust, and worthy when applied to discover low individual QTN effect values for quality traits in rice.

Several rice grain quality genes, such as *Badh2*, *DEP2*, *GW7*, *OsCESA2*, and *OsCPK8*, have been functionally characterized over the past 10 years (Deveshwar et al., 2020; Imran et al., 2022; Yan et al., 2022). Among these, *Badh2*, the *fgr* gene, the major gene causing fragrance in rice and a frameshift mutation in its exonic region, is the functional allele associated with fragrance (Quero et al., 2018; Tibbs et al., 2021). *DEP2/SRS1* encoding the dense and erect panicle 2 gene positively regulates panicle morphology and its outgrowth, suggesting its direct role in regulating rice grain size and yield at the genetic level (Li et al., 2010). *GW7* is annotated as a gene encoding a TONNEAU1-recruiting motif protein that simultaneously controls grain width and quality (Li et al., 2010).

Combining the cloned genes/QTLs reported in earlier genetic studies, 19 QTNs and their ±100 kb genomic regions superimposed the previously annotated grain-quality genes. QTNs clusters were mapped for HRR (%) on chromosome 7 (*qHRR-7-1*, *qHRR-7-2*, and *qHRR-7-3*) located in the vicinity of *GW7* and *DEP2*, which are responsible for grain yield and quality, and another cluster was detected on chromosome 11 (*qHRR-11-1*, *qHRR-11-2*, *qHRR-11-3*, and *qHRR-11-4*) near the F-box and DUF domain-containing genes with confirmed roles in improving yield potential and quality in rice. Additionally, 20 novel QTNs were excluded from the genomic loci of earlier studies, and the markers detected may be the putative QTNs governing quality traits in rice.

### Dissecting four candidate genes of grain quality traits

Using multiple models for association studies, three QTNs (*qAR-1-2*, *qHRR-7-2*, and *qPGC-2-3*) were confirmed to have major gene effects on grain quality. The candidate region of 38.37 Mb to 38.42 Mb in qAR-1-2 was fine-mapped considering a threshold value of r^2^ >0.2 (**Figure 6A**). Five genes located in this genomic region were possible candidates governing aroma in rice, and haplotyping was performed for each of the five genes. Significant differences in aroma scores between the *LOC_Os01g66110* and *LOC_Os01g66140* haplotypes were observed (**Figure 6B**). *LOC_Os01g66110*, a putative methyltransferase, has been proposed to play a role in multiple epigenomic modifications of heavy-metals transporters involved in the 2-AP biosynthesis pathway. In recent years, the occurrence of DNA methylation of all types (CHH, CHG, and CG) in genes related to 2-AP biosynthesis has been reported in rice. ChIP-seq, bisulfite-seq, and ATAC-seq data of aroma genes also showed active chromatin modifications as key regulators (Imran et al., 2022) with strong enrichment of H3K36me3 at 2-AP biosynthesis pathway-related genes. Another candidate gene, *LOC_Os01g66140*, annotated as a plus-3 domain-containing protein, is anticipated to influence 2-AP biosynthesis genes with metal-binding properties and DNA-binding domains. BLAST tool and STRING analysis revealed that *LOC_Os01g66140* directly interacts with histone H4 and zinc metal ions, confirming its role in regulating 2-AP content in aromatic rice. Prior studies have found that exogenous application of micronutrients, specifically zinc, could upregulate genes involved in the biosynthesis of 2-AP in aromatic rice due to increased levels of proline and proline dehydrogenase (He and Park, 2015). Based on these findings, we propose that *LOC_Os01g66110* and *LOC_Os01g66140* may be related to the grain aroma. Their role in regulating heavy metal transporters in response to zinc is worthy of comprehensive studies and confirmation.

The candidate *qHRR-7-2* associated with HRR (%), *LOC_Os07g44910*, annotated as the gibberellin receptor *GID1L2*, is a type of F-box subunit of the S-phase kinase-associated protein 1 (SKP1)-cullin 1 (CUL1)-F-box protein (SCF) E3 complex that encodes the alpha/beta hydrolase fold-3 domain-containing protein containing 358 amino acids, belonging to the alpha/beta hydrolase (ABH) superfamily. The F-box protein (SCF) E3 complex plays a crucial role in regulating life processes such as cell division and influences grain size and yield in rice by facilitating proteasomal degradation of diverse regulatory proteins (Chen et al., 2008; Nguyen and Busino, 2020). Its loss-of-function mutants, htd4 and dta-34 have reduced panicle branching, grains/panicle, and seed size, and show a dwarf phenotype (Wang et al., 2017; Liang et al., 2019, Liu et al, 2009). For instance, *Grain weight 2* (*GW2*), encoding E3 ubiquitin ligase, regulates grain weight and grain yield by ubiquitinating *EXPLA 1* and promoting its degradation (Hu et al., 2015; Mo et al., 2016; Deveshwar et al., 2020). In this study, GWAS and haplotype analysis results indicated that *LOC_Os07g44910* might govern grain weight and grain yield in rice (**Figures 7A, B**). Members of this superfamily, such as *GS5* (*Grain Size 5*, putative serine carboxypeptidase) (Hu et al., 2015) and *TGW6* (*Thousand Grain Weight 6*, IAA-glucose hydrolase) (Mo et al., 2016), have been characterized for their roles in influencing grain weight and yield. These studies showed high consistency with our GWAS analysis results, confirming with these printed reports proving that *LOC_Os07g44910* might be related to rice recovery % (HRR, %) in rice.

In the candidate *qPGC-2-3*, involved in the percentage of grains with chalkiness, *LOC_Os02g14120* is a Brassinosteriod Insensitive 1 Associated Receptor Kinase 1 precursor gene (*OsBAK 1-9*). *OsBAK1*/*Top Bending Panicle 1* encodes a somatic embryogenesis receptor kinase (SERK) domain-containing protein that acts as a modulating factor in the brassinosteroid signaling pathway, thus affecting the number of grains and yield in rice (Xing and Zhang, 2010; Gupta et al., 2022). Overexpression of OsBAK-1 drastically reduced grain yield in rice (Lin et al., 2017), and its high-tillering mutants are characterized by a reduction in panicle length and seed size (Deveshwar et al., 2020). The central role of brassinosteroids (BR) in regulating multiple biological processes such as flowering, male fertility, and tillering, is becoming more apparent (Lin et al., 2017; Yuan et al., 2017). Although, brassinosteroids have been demonstrated to be positive regulators of plant growth processes and grain development, they most often work in close association with auxins and cytokinins to affect the efficiency of photosynthesis, sugar metabolism, and mobilizing resources in crop plants to influence grain filling (Mo et al., 2016; Deveshwar et al., 2020), reiterating the need to consider the holistic approach of plant developmental processes and their architecture to improve crop yields. These results suggest that *LOC_Os02g14120* may be related to the percentage of grains with chalkiness (PGC, %), and its role in modulating the architecture, yield, and grain quality in rice is valuable for further evaluation and validation.

## Conclusions

In this GWAS analysis, 70 QTNs were detected for three grain quality traits using different multi-locus methodologies. Among these QTNs, *qAR-1-2*, *qHRR-7-2*, and *qPGC-2-3*, which are closely associated with AR, HRR (%), and PGC (%), were identified using both single- and multi-locus methods. In addition, four key annotated genes (*LOC_Os01g66110*, *LOC_Os01g66140*, *LOC_Os07g44910*, and *LOC_Os02g14120*) that govern the three target candidate genes mentioned above were mined. In conclusion, several robust QTLs and four candidate functional genes were shown to possibly control grain aroma, head rice recovery (%), and the percentage of grains with chalkiness in rice. This investigation provides valuable information for functional characterization in the future and molecular marker-based breeding design to improve appearance quality traits in rice.

## Data availability statement

The original contributions presented in the study are included in the article/**Supplementary Files**, further inquiries can be directed to the corresponding author/s.

## Author contributions

SS: Data curation, Formal Analysis, Methodology, Software, Validation, Writing – original draft. RS: Conceptualization, Funding acquisition, Investigation, Project administration, Resources, Supervision, Visualization, Writing – review & editing. AM: Formal Analysis, Software, Writing – review & editing. VS: Writing – review & editing. US: Resources, Writing – review & editing. AK: Writing – review & editing. GS: Writing – review & editing.
