# Supplementary material for: Multi-model genome-wide association studies for appearance quality in rice
Source: Front Plant Sci. 2024 Jan 11;14:1304388. doi: 10.3389/fpls.2023.1304388 (PMC10808671; doi:10.3389/fpls.2023.1304388)
Supplement: Supplementary file 1 [file DataSheet_1.docx]

Supplementary Material

**Supplementary Figure 1:** Distribution of quality related grain QTNs identified using the three multi-locus GWAS models.

#
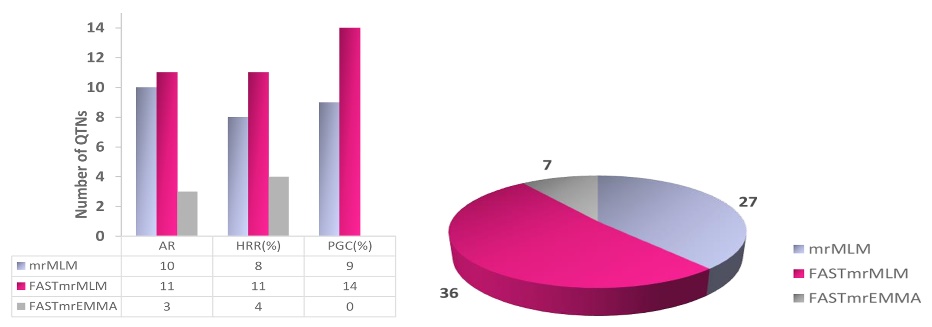


**Supplementary Table 1:** Details of 196 rice accessions used in the present study.

| **Name** | **Subpopulation*** | **Genetic stock_vername** | **Country source** |
| --- | --- | --- | --- |
| IRIS 313-9557 | ind2 | MULLIKURUVA::IRGC 77529-1-1 | India |
| IRIS 313-10403 | indx | IRGA 659-1-2-2-2::IRGC 117345-1-1 | Colombia |
| IRIS 313-9218 | ind2 | KALO CHAKOL::IRGC 77258-1-1 | Bangladesh |
| IRIS 313-9778 | admix | GENIT::IRGC 3272-1-1 | Argentina |
| IRIS 313-10170 | ind1A | MIN ZAO 6::IRGC 63772-1-1 | China |
| IRIS 313-11957 | indx | DAW LUEY 4-3::IRGC 76929-1 | Thailand |
| IRIS 313-10352 | indx | CT 9737-6-1-1-2-2P-M::IRGC 117330-1-1 | Colombia |
| IRIS 313-10148 | indx | CAUVERY::IRGC 45255-1-1 | India |
| IRIS 313-9019 | ind3 | KAM PAI::IRGC 78245-1-1 | Thailand |
| IRIS 313-10226 | indx | TONG GU HONG::IRGC 81026-1-1 | China |
| IRIS 313-9072 | ind2 | MUTA GANJE::IRGC 26744-1-1 | Bangladesh |
| IRIS 313-10179 | indx | KAMRANGA::IRGC 26373-2 | China |
| IRIS 313-7736 | ind2 | NONA BOKRA::IRGC 22710-C1-G1 | India |
| IRIS 313-8721 | aus | HOLOI BASH (SOLOI BASH)::IRGC 64778-1-1 | Bangladesh |
| IRIS 313-10361 | indx | IR 63295-AC 209-7::IRGC 117365-1-1 | Philippines |
| IRIS 313-9422 | aus | CHUNGUR BALI::IRGC 25855-1-1 | Bangladesh |
| IRIS 313-10334 | ind1B | B 6136 E 3-TB-0-1-5::IRGC 117311-1-1 | Indonesia |
| IRIS 313-10809 | trop | GAO JIAO HONG::IRGC 80920-1 | Indonesia |
| IRIS 313-11905 | ind3 | QINOGNAS::IRGC 8135-1 | Indonesia |
| IRIS 313-10341 | indx | BR 5230-46-4::IRGC 117318-1-1 | Bangladesh |
| IRIS 313-9384 | ind2 | BARIK KUDI::IRGC 52807-1-1 | India |
| IRIS 313-7780 | indx | SONA::IRGC 26971-C1-G1 | India |
| IRIS 313-11431 | ind3 | C 166-135::IRGC 50633-1 | Philippines |
| IRIS 313-11078 | indx | NON HAI::IRGC 29636-1 | Laos |
| IRIS 313-11902 | ind3 | CEMPO TURI::IRGC 73667-1 | Indonesia |
| IRIS 313-11242 | ind2 | OR 117-8::IRGC 39680-1 | India |
| IRIS 313-9925 | ind1B | MILYANG 30::IRGC 46977-1-1 | South Korea |
| IRIS 313-9966 | ind1B | CICA 9::IRGC 53079-1-1 | Colombia |
| IRIS 313-8341 | indx | BAT DO::IRGC 7014-1-1 | Vietnam |
| IRIS 313-9730 | ind1A | BA SHI ZAO::IRGC 67903-1-1 | China |
| IRIS 313-8660 | ind2 | KOTTEYARAN::IRGC 47383-1-1 | Sri Lanka |
| IRIS 313-11384 | indx | CHIEM TONG NHAT 1::IRGC 47496-1 | Vietnam |
| IRIS 313-9572 | indx | ASU::IRGC 62154-1-1 | Bhutan |
| IRIS 313-10400 | ind1B | IRGA 370-38-1-1F-C4-2::IRGC 117342-1-1 | Colombia |
| IRIS 313-10899 | ind3 | DAMNOEUB KHSE SAUT::IRGC 22819-1 | Cambodia |
| IRIS 313-10300 | indx | TANALA::IRGC 79467-1 | Colombia |
| IRIS 313-10002 | ind1B | BW 295-5::IRGC 63098-1-1 | Sri Lanka |
| IRIS 313-10040 | ind1B | MILYANG 77::IRGC 69340-1-1 | South Korea |
| IRIS 313-11056 | aus | AUS 301::IRGC 29089-1 | Bangladesh |
| IRIS 313-9822 | ind1A | CRILLO LA FRIA::IRGC 10793-1-1 | Venezuela |
| IRIS 313-8414 | indx | ARC 18202::IRGC 42328-1-1 | India |
| IRIS 313-9629 | aro | JC 157::IRGC 9074-1-1 | India |
| IRIS 313-8940 | ind1A | FU ZAO XIAN::IRGC 63619-1-1 | China |
| IRIS 313-10811 | indx | SEMPOR::IRGC 18761-1 | Indonesia |
| IRIS 313-8679 | ind3 | JAO LEUANG::IRGC 65866-1-1 | Thailand |
| IRIS 313-9201 | trop | ARC 6044::IRGC 12190-1-1 | India |
| IRIS 313-10046 | ind1B | CHAMA (DWARF)::IRGC 69487-1-1 | Zambia |
| IRIS 313-10177 | indx | DA GANG ZHAN::IRGC 67103-1-1 | China |
| IRIS 313-8454 | ind1A | LAI YIP ZIM::IRGC 4955-1-1 | Taiwan |
| IRIS 313-8932 | ind3 | PATISAIL::IRGC 37562-1-1 | Bangladesh |
| IRIS 313-8924 | ind2 | KUTTA::IRGC 52184-1-1 | India |
| IRIS 313-11423 | indx | C 1016-1::IRGC 50368-1 | Philippines |
| IRIS 313-11059 | aus | AUS 344::IRGC 29131-1 | Bangladesh |
| IRIS 313-10167 | indx | IR 13429-109-2-2-1::IRGC 63491-1-1 | Philippines |
| IRIS 313-9137 | aus | ARC 10100::IRGC 20709-1-1 | India |
| IRIS 313-9706 | ind1A | TAIPEI WOO CO::IRGC 112-1-1 | Taiwan |
| IRIS 313-12246 | ind3 | BANGKOUY::IRGC 94037-1 | Cambodia |
| IRIS 313-10620 | trop | BOW SU SO::IRGC 78237-1 | Brazil |
| IRIS 313-9522 | indx | RPW 9-4 (SS 1)::IRGC 50690-1-1 | India |
| IRIS 313-9368 | aus | CHANDARHAT::IRGC 25845-1-1 | Bangladesh |
| IRIS 313-11048 | aus | AUS 171::IRGC 29004-1 | Bangladesh |
| IRIS 313-10271 | indx | IA CUBA 17::IRGC 116990-1-1 | Cuba |
| IRIS 313-10996 | indx | BAKASI::IRGC 27074-1 | Indonesia |
| IRIS 313-10026 | ind2 | KITRANA 1007::IRGC 68517-1-1 | Madagascar |
| IRIS 313-12039 | ind3 | ITA 131::IRGC 80025-1 | Cambodia |
| IRIS 313-8412 | indx | FEI GAI 122::IRGC 63599-1-1 | China |
| IRIS 313-9758 | ind1A | I KUNG PAO::IRGC 114-1-1 | Taiwan |
| IRIS 313-8586 | ind3 | PLI KHAO::IRGC 64596-1-1 | Thailand |
| IRIS 313-10171 | ind1A | YA NONG ZAO 4::IRGC 63908-1-1 | China |
| IRIS 313-9935 | ind2 | MEKENZIE SMALL::IRGC 49895-1-1 | Guyana |
| IRIS 313-10940 | ind3 | J 6 IR 520 (WC 693)::IRGC 57600-1 | Indonesia |
| IRIS 313-8812 | ind3 | RELLY::IRGC 14623-1-1 | Indonesia |
| IRIS 313-11615 | ind3 | MANARE (PHOM)::IRGC 61437-1 | Guinea |
| IRIS 313-8305 | ind2 | URAIBOOL::IRGC 52785-1-1 | India |
| IRIS 313-10401 | ind1B | IRGA 370-42-1-1F-C-1::IRGC 117343-1-1 | Colombia |
| IRIS 313-8731 | ind2 | NIBARI::IRGC 67742-1-1 | India |
| IRIS 313-8530 | ind2 | DHANE BURWA::IRGC 10105-1-1 | India |
| IRIS 313-9391 | ind2 | KALABAIL::IRGC 25877-1-1 | Bangladesh |
| IRIS 313-10129 | ind1A | SAN SHIH TSI::IRGC 1038-1-1 | China |
| IRIS 313-11517 | ind1B | IR 4432-28-5::IRGC 55952-1 | Philippines |
| IRIS 313-10151 | ind3 | RD 15::IRGC 47705-1-1 | Thailand |
| IRIS 313-9259 | ind2 | G 25::IRGC 45733-1-1 | India |
| IRIS 313-9032 | ind1B | KHAO GRADOOK CHAHNG::IRGC 17111-1-1 | Thailand |
| IRIS 313-11054 | aus | AUS 295::IRGC 29083-1 | Bangladesh |
| IRIS 313-10257 | indx | ALTAMIRA 9::IRGC 116953-1-1 | Nicaragua |
| IRIS 313-10983 | indx | BOROJYOT::IRGC 26578-1 | Bangladesh |
| IRIS 313-9469 | ind1A | TSAO SHENG LI 1::IRGC 1309-1-1 | China |
| IRIS 313-8641 | aus | AUS 439::IRGC 29221-1-1 | Bangladesh |
| IRIS 313-8900 | ind2 | ARC 14654::IRGC 41663-1-1 | India |
| IRIS 313-11588 | ind3 | MR 69::IRGC 60188-1 | Malaysia |
| IRIS 313-10279 | indx | IR 3839-1::IRGC 55946-1 | Dominican Republic |
| IRIS 313-9732 | ind2 | MAMORIAKA::IRGC 68672-1-1 | Madagascar |
| IRIS 313-12040 | ind3 | NEANG LAU::IRGC 81328-1 | Cambodia |
| IRIS 313-8914 | indx | E 2040::IRGC 67968-1-1 | China |
| IRIS 313-10736 | aus | RERM BILASH::IRGC 16273-1 | Nepal |
| IRIS 313-8963 | aus | BATHURI::IRGC 25838-1-1 | Bangladesh |
| IRIS 313-9066 | ind1B | KULA KARUPPAN::IRGC 55328-1 | Bangladesh |
| IRIS 313-10333 | ind1B | B 6136-3-TB-0-1-5::IRGC 117312-1-1 | Indonesia |
| IRIS 313-10221 | indx | BA BAI GU::IRGC 79580-1-1 | China |
| IRIS 313-9634 | indx | ARC 15873::IRGC 43250-1-1 | India |
| IRIS 313-8968 | ind2 | KALU ILANKALAYAN::IRGC 36270-1-1 | Sri Lanka |
| IRIS 313-10189 | ind1A | DA NUO (ZHAN)::IRGC 72025-1-1 | China |
| IRIS 313-9626 | aus | KALIA::IRGC 34699-1-1 | Bangladesh |
| IRIS 313-10287 | ind1B | UQUIHUA::IRGC 117037-1-1 | Peru |
| IRIS 313-9429 | indx | RTS 16::IRGC 8235-1-1 | Vietnam |
| IRIS 313-11515 | indx | BKN BR 1031-78-5-4::IRGC 55927-1 |  |
| IRIS 313-8846 | indx | BAIANG 6::IRGC 6129-1-1 | Indonesia |
| IRIS 313-8699 | indx | BALASURIYA A::IRGC 66509-1-1 | Sri Lanka |
| IRIS 313-10237 | ind1B | PSBRC 86::IRGC 99716-1-1 | Philippines |
| IRIS 313-8994 | indx | ARC 14064::IRGC 41377-1-1 | India |
| IRIS 313-8930 | ind1B | MUKKALA BAZAL::IRGC 77279-1-1 | Bangladesh |
| IRIS 313-9403 | ind2 | BADUIE::IRGC 53715-1-1 | India |
| IRIS 313-8405 | indx | JIN JUN DAO::IRGC 59710-1-1 | China |
| IRIS 313-9551 | ind2 | BENGALY MORIMO::IRGC 10976-1-1 | Madagascar |
| IRIS 313-10294 | indx | IR 21015-72-3-3-3-1::IRGC 117004-1-1 | Philippines |
| IRIS 313-8793 | ind3 | KHAO THI RATE::IRGC 58041-1-1 | Myanmar |
| IRIS 313-10842 | ind3 | LUBUK LINGGAU::IRGC 20000-1 | Indonesia |
| IRIS 313-9611 | ind2 | WANGA BARUGULU::IRGC 52261-1-1 | India |
| IRIS 313-11692 | ind1A | CHIAYI WU-K'O::IRGC 64974-1 | Taiwan |
| IRIS 313-9924 | indx | KN 1 B 361-1-8-6-9::IRGC 46974-1-1 | South Korea |
| IRIS 313-10020 | aus | HODARAWALA::IRGC 67631-1-1 | Sri Lanka |
| IRIS 313-8603 | indx | ARC 12884::IRGC 22417-1-1 | India |
| IRIS 313-10274 | ind1B | ICTA CRISPO 38::IRGC 116994-1-1 | Guatemala |
| IRIS 313-8437 | indx | IRRIBINI::IRGC 49094-1-1 | Bangladesh |
| IRIS 313-10298 | ind1B | 3210::IRGC 116950-1-1 | Sri Lanka |
| IRIS 313-8789 | aus | LALSAITA::IRGC 43915-1-1 | Bangladesh |
| IRIS 313-8568 | ind2 | LARHA MUGAD::IRGC 52339-1-1 | India |
| IRIS 313-12043 | ind3 | SAMBOK ANGKRANG::IRGC 81375-1 | Cambodia |
| IRIS 313-11049 | aus | AUS 219::IRGC 29031-1 | Bangladesh |
| IRIS 313-9111 | ind1A | XIA ZHI BAI::IRGC 53437-1-1 | China |
| IRIS 313-10497 | ind1A | CE IN TSAN::IRGC 4362-1 | China |
| IRIS 313-8697 | ind3 | YEBAWYIN::IRGC 33885-1-1 | Myanmar |
| IRIS 313-8727 | ind2 | T 315::IRGC 54792-1-1 | India |
| IRIS 313-10290 | ind1B | ELONI::IRGC 116980-1-1 | Surinam |
| IRIS 313-11745 | ind1A | AN FU ZHAN::IRGC 67878-1 | China |
| IRIS 313-9944 | indx | SOLOMON RED RICE::IRGC 50950-1-1 | Solomon Islands |
| IRIS 313-8980 | ind3 | E DAW HAWM::IRGC 47938-1-1 | Thailand |
| IRIS 313-8559 | ind2 | KEERIPALA CHILL PADDY::IRGC 49790-1-1 | India |
| IRIS 313-10337 | indx | B 6149 F-MR-7::IRGC 117314-1-1 | Indonesia |
| IRIS 313-8854 | ind2 | CHAKOL::IRGC 77226-1-1 | Bangladesh |
| IRIS 313-9970 | ind2 | RACE PERUMAL::IRGC 55347-1-1 | Sri Lanka |
| IRIS 313-9400 | indx | NCS 964 C::IRGC 62604-1-1 | India |
| IRIS 313-11355 | indx | CN 44-40-7::IRGC 45368-1 | India |
| IRIS 313-10301 | indx | IRGA 959-1-2-2F-4-1-4A-6-CA-6X::IRGC 117006-1-1 | Brazil |
| IRIS 313-10178 | ind1A | GAO JIAO BAI::IRGC 68047-1-1 | China |
| IRIS 313-11164 | aus | BAK TULSI::IRGC 34831-1 | India |
| IRIS 313-9953 | indx | JHONA 101::IRGC 27976-1 | Mexico |
| IRIS 313-8386 | indx | ARC 10812::IRGC 21074-1-1 | India |
| IRIS 313-9917 | ind1B | CHANDINA::IRGC 36420-1-1 | Sri Lanka |
| IRIS 313-8492 | ind3 | KUNENG::IRGC 71545-1-1 | Malaysia |
| IRIS 313-8903 | ind3 | BPI 76 NON SENSITIVE (GREEN)::IRGC 9790-1-1 | Philippines |
| IRIS 313-8725 | ind3 | PULUT BARAYA::IRGC 27393-1-1 | Indonesia |
| IRIS 313-10161 | ind1B | BR IRGA 409::IRGC 55915-1-1 | Brazil |
| IRIS 313-8796 | ind2 | DUDH KADAR::IRGC 67707-1-1 | India |
| IRIS 313-7690 | indx | IR 2344-P1 PB-9-3-2B::IRGC 39317-C1-G1 | Philippines |
| IRIS 313-10690 | ind3 | TELURIKAN::IRGC 27478-1 | Indonesia |
| IRIS 313-10480 | indx | PULUT NANGKA 16::IRGC 3630-1 | Indonesia |
| IRIS 313-8571 | ind3 | ES 21::IRGC 56171-1-1 | Tanzania |
| IRIS 313-10307 | indx | INIAP 6::IRGC 117002-1-1 | Ecuador |
| IRIS 313-11057 | aus | AUS 308::IRGC 29096-1 | Bangladesh |
| IRIS 313-8982 | indx | ARC 18112::IRGC 42274-1-1 | India |
| IRIS 313-10527 | ind2 | ADT 12::IRGC 6254-1 | India |
| IRIS 313-9989 | ind3 | MELEKE::IRGC 56823-1-1 | Ivory Coast |
| IRIS 313-8986 | indx | ARC 10754::IRGC 12603-1-1 | India |
| IRIS 313-8585 | indx | ARC 11901::IRGC 21727-1-1 | India |
| IRIS 313-11326 | indx | BR 51-115-4::IRGC 43999-1 | Bangladesh |
| IRIS 313-10394 | indx | IR 80310-12-B-1-3-B::IRGC 117307-1-1 | Philippines |
| IRIS 313-9139 | ind2 | GOJOL GORIA::IRGC 26629-1-1 | Bangladesh |
| IRIS 313-9065 | ind1A | PAI CHUEH CHIU LIU::IRGC 34259-1-1 | China |
| IRIS 313-9922 | ind1B | IRI 339::IRGC 46956-1-1 | South Korea |
| IRIS 313-9505 | ind1A | KORASISI::IRGC 5285-1-1 | Philippines |
| IRIS 313-11138 | ind3 | NGASEIN THEEDAT (C 30)::IRGC 33504-1 | Myanmar |
| IRIS 313-10392 | ind1B | IR 77390-1-6-4-19-1-B::IRGC 117303-1-1 | Philippines |
| IRIS 313-8244 | ind2 | POKKALI::IRGC 8948-1-1 | Sri Lanka |
| IRIS 313-10041 | trop | BOTOHAVANA MENA::IRGC 69349-1-1 | Madagascar |
| IRIS 313-9262 | ind2 | JHODI BIRUN::IRGC 31812-1-1 | Bangladesh |
| IRIS 313-9841 | indx | PULU RENNI::IRGC 27386-1 | Surinam |
| IRIS 313-11044 | trop | ARC 18502::IRGC 51749-2 | Malaysia |
| IRIS 313-10035 | ind2 | RIZ TYPE SORGHO::IRGC 69015-1-1 | Madagascar |
| IRIS 313-11555 | indx | PA WOON::IRGC 58656-1 | Sierra Leone |
| IRIS 313-10385 | ind1B | IR 75870-5-8-5-B-1::IRGC 117297-1-1 | Philippines |
| IRIS 313-11921 | ind3 | KHAO GAHB BUA::IRGC 74937-1 | Thailand |
| IRIS 313-9115 | ind3 | LEUANG YAI 29-12-2::IRGC 881-1-1 | Thailand |
| IRIS 313-11052 | aus | AUS 278::IRGC 29068-1 | Bangladesh |
| IRIS 313-10652 | ind3 | CHAO PEUAK DENG::IRGC 11602-1 | Laos |
| IRIS 313-9227 | ind1B | WP 65::IRGC 36526-1-1 | Thailand |
| IRIS 313-11140 | ind3 | PADAN::IRGC 33544-1 | Myanmar |
| IRIS 313-11058 | aus | AUS 329::IRGC 29116-1 | Bangladesh |
| IRIS 313-8743 | indx | NIAO YAO::IRGC 5496-1-1 | Taiwan |
| IRIS 313-10000 | ind1B | SUWEON 311::IRGC 61890-1-1 | South Korea |
| IRIS 313-10374 | indx | IR 69502-6-SRN-3-UBN-1-B::IRGC 117290-1-1 | Philippines |
| IRIS 313-10768 | ind3 | GADABUNG (GUNDIL)::IRGC 17569-1 | Indonesia |
| IRIS 313-8645 | ind1A | PAI YI PING::IRGC 1368-1-1 | China |
| IRIS 313-10268 | indx | FONAIAP 2::IRGC 116985-1-1 | Venezuela |
| IRIS 313-11010 | ind3 | PULUT PUTIH::IRGC 27426-1 | Indonesia |
| IRIS 313-9433 | ind2 | GOKULGANJA::IRGC 45701-1-1 | India |

*ind1A, ind1B, ind2 and ind3 are three groups of indica rice, indx corresponds to other indica varieties, japx is other japonica varieties, aus is aus, aro is aromatic and admix is all other unassigned varieties (<http://www.gigasciencejournal.cm/content/3/1/7>).

**Supplementary Table 2:** Descriptive statistics for three grain quality traits of rice.

| **Traits** | **Mean ± SD** | **Range** | **skewness** | **Kurtosis** | **CV (%)** | **Heritability (H^2^)** |
| --- | --- | --- | --- | --- | --- | --- |
| Grain aroma | 0.15 ± 0.48 | 0-3 | 3.38 | 11.58 | 3.16 | 0.28 |
| Head rice recovery (%) | 23.68 ± 14.8 | 5.05-64.69 | 0.98 | 0.17 | 0.63 | 0.99 |
| Percent with grain chalkiness (%) | 28.45 ± 22.21 | 0-100 | 1.36 | 2.01 | 0.78 | 0.98 |

Where CV: Coefficient of variation (%)

H^2^: Broad-sense Heritability

**Supplementary Table 3:** SNPs and QTLs mapping information in MLM analysis.

| **Table 3-1** SNPs mapping information of MLM analysis. | | | | | |  |  |  |
| --- | --- | --- | --- | --- | --- | --- | --- | --- |
| **Trait** | **SNP** | **Chr** | **Position** | **P.value** | **MAF** | **R square. of.**  **Model.**  **with. SNP** | **effect** | **R^2^(%)** |
| AR | 1081413 | 1 | 1081413 | 0.00 | 0.129 | 0.099 | -0.231 | 0.000 |
| AR | 1085222 | 1 | 1085222 | 0.00 | 0.177 | 0.074 | -0.168 | 0.001 |
| AR | 1085958 | 1 | 1085958 | 0.00 | 0.134 | 0.093 | -0.217 | 0.190 |
| AR | 20227999 | 1 | 20227999 | 0.00 | 0.071 | 0.129 | -0.386 | 0.003 |
| AR | 24236941 | 1 | 24236941 | 0.00 | 0.051 | 0.076 | 0.309 | 0.040 |
| AR | 2425519 | 1 | 2425519 | 0.00 | 0.073 | 0.069 | -0.307 | 0.000 |
| AR | 32391762 | 1 | 32391762 | 0.00 | 0.083 | 0.090 | -0.277 | 3.013 |
| AR | 3444839 | 1 | 3444839 | 0.00 | 0.356 | 0.068 | 0.149 | 0.000 |
| AR | 36249172 | 1 | 36249172 | 0.00 | 0.078 | 0.083 | -0.283 | 0.041 |
| AR | 36309267 | 1 | 36309267 | 0.00 | 0.083 | 0.077 | 0.266 | 0.000 |
| AR | 37798495 | 1 | 37798495 | 0.00 | 0.081 | 0.069 | 0.251 | 1.822 |
| AR | 38093726 | 1 | 38093726 | 0.00 | 0.063 | 0.069 | 0.274 | 0.158 |
| AR | 38383904 | 1 | 38383904 | 0.00 | 0.056 | 0.070 | 0.275 | 3.371 |
| AR | 41075437 | 1 | 41075437 | 0.00 | 0.179 | 0.076 | -0.178 | 0.000 |
| AR | 41079399 | 1 | 41079399 | 0.00 | 0.177 | 0.078 | -0.183 | 0.000 |
| AR | 41105276 | 1 | 41105276 | 0.00 | 0.051 | 0.071 | 0.292 | 0.000 |
| AR | 41110769 | 1 | 41110769 | 0.00 | 0.051 | 0.071 | -0.292 | 0.000 |
| AR | 304255342 | 10 | 10770072 | 0.00 | 0.073 | 0.070 | 0.242 | 0.000 |
| AR | 306473354 | 10 | 12988084 | 0.00 | 0.184 | 0.069 | -0.167 | 0.000 |
| AR | 307317512 | 10 | 13832242 | 0.00 | 0.091 | 0.068 | -0.398 | 0.000 |
| AR | 307546356 | 10 | 14061086 | 0.00 | 0.053 | 0.086 | 0.347 | 0.000 |
| AR | 307774564 | 10 | 14289294 | 0.00 | 0.124 | 0.093 | -0.256 | 0.018 |
| AR | 309558735 | 10 | 16073465 | 0.00 | 0.157 | 0.083 | -0.279 | 0.000 |
| AR | 309561009 | 10 | 16075739 | 0.00 | 0.157 | 0.083 | 0.279 | 0.000 |
| AR | 309561378 | 10 | 16076108 | 0.00 | 0.164 | 0.109 | 0.311 | 0.000 |
| AR | 309567327 | 10 | 16082057 | 0.00 | 0.157 | 0.083 | 0.279 | 1.891 |
| AR | 309659291 | 10 | 16174021 | 0.00 | 0.167 | 0.118 | 0.322 | 0.014 |
| AR | 309663862 | 10 | 16178592 | 0.00 | 0.169 | 0.082 | -0.252 | 0.031 |
| AR | 310750457 | 10 | 17265187 | 0.00 | 0.051 | 0.118 | -0.379 | 0.000 |
| AR | 310793887 | 10 | 17308617 | 0.00 | 0.051 | 0.118 | -0.379 | 0.015 |
| AR | 310880751 | 10 | 17395481 | 0.00 | 0.056 | 0.082 | -0.303 | 0.000 |
| AR | 310897504 | 10 | 17412234 | 0.00 | 0.061 | 0.095 | 0.311 | 0.024 |
| AR | 314820179 | 10 | 21334909 | 0.00 | 0.053 | 0.080 | -0.325 | 0.725 |
| AR | 315654192 | 10 | 22168922 | 0.00 | 0.199 | 0.072 | -0.168 | 0.057 |
| AR | 316132298 | 10 | 22647028 | 0.00 | 0.051 | 0.092 | -0.362 | 0.000 |
| AR | 316571966 | 10 | 23086696 | 0.00 | 0.139 | 0.086 | -0.203 | 0.000 |
| AR | 316603324 | 10 | 23118054 | 0.00 | 0.129 | 0.071 | 0.189 | 0.025 |
| AR | 316624792 | 10 | 23139522 | 0.00 | 0.154 | 0.075 | -0.181 | 0.022 |
| AR | 316667568 | 10 | 23182298 | 0.00 | 0.129 | 0.071 | 0.189 | 0.003 |
| AR | 296031111 | 10 | 2545841 | 0.00 | 0.056 | 0.133 | -0.382 | 0.000 |
| AR | 296092912 | 10 | 2607642 | 0.00 | 0.088 | 0.093 | -0.297 | 0.000 |
| AR | 296094498 | 10 | 2609228 | 0.00 | 0.096 | 0.072 | -0.230 | 0.202 |
| AR | 296147476 | 10 | 2662206 | 0.00 | 0.051 | 0.105 | 0.363 | 0.000 |
| AR | 296156618 | 10 | 2671348 | 0.00 | 0.051 | 0.073 | 0.308 | 0.252 |
| AR | 296467858 | 10 | 2982588 | 0.00 | 0.051 | 0.069 | -0.282 | 0.294 |
| AR | 293816702 | 10 | 331432 | 0.00 | 0.071 | 0.083 | 0.274 | 0.432 |
| AR | 297083360 | 10 | 3598090 | 0.00 | 0.051 | 0.069 | -0.282 | 0.000 |
| AR | 297470061 | 10 | 3984791 | 0.00 | 0.081 | 0.091 | 0.266 | 1.508 |
| AR | 297564129 | 10 | 4078859 | 0.00 | 0.179 | 0.073 | -0.174 | 0.000 |
| AR | 298246846 | 10 | 4761576 | 0.00 | 0.157 | 0.076 | -0.247 | 4.549 |
| AR | 301451349 | 10 | 7966079 | 0.00 | 0.149 | 0.070 | 0.277 | 0.000 |
| AR | 301671332 | 10 | 8186062 | 0.00 | 0.149 | 0.075 | 0.302 | 0.000 |
| AR | 302145468 | 10 | 8660198 | 0.00 | 0.154 | 0.071 | 0.286 | 0.000 |
| AR | 302424886 | 10 | 8939616 | 0.00 | 0.154 | 0.071 | 0.286 | 0.032 |
| AR | 339225043 | 11 | 22532486 | 0.00 | 0.098 | 0.069 | -0.205 | 0.000 |
| AR | 342679293 | 11 | 25986736 | 0.00 | 0.192 | 0.092 | -0.213 | 0.000 |
| AR | 316963484 | 11 | 270927 | 0.00 | 0.303 | 0.069 | 0.160 | 0.000 |
| AR | 316966296 | 11 | 273739 | 0.00 | 0.199 | 0.074 | -0.169 | 0.962 |
| AR | 344170124 | 11 | 27477567 | 0.00 | 0.154 | 0.081 | 0.187 | 0.000 |
| AR | 317005458 | 11 | 312901 | 0.00 | 0.194 | 0.093 | -0.196 | 0.341 |
| AR | 317009574 | 11 | 317017 | 0.00 | 0.275 | 0.070 | 0.176 | 0.000 |
| AR | 317012795 | 11 | 320238 | 0.00 | 0.275 | 0.071 | 0.181 | 0.000 |
| AR | 317018300 | 11 | 325743 | 0.00 | 0.275 | 0.073 | -0.187 | 0.000 |
| AR | 317021021 | 11 | 328464 | 0.00 | 0.275 | 0.073 | 0.186 | 0.000 |
| AR | 317023180 | 11 | 330623 | 0.00 | 0.270 | 0.073 | -0.184 | 0.000 |
| AR | 317035464 | 11 | 342907 | 0.00 | 0.273 | 0.076 | -0.193 | 0.000 |
| AR | 317037912 | 11 | 345355 | 0.00 | 0.273 | 0.076 | 0.193 | 0.000 |
| AR | 317040423 | 11 | 347866 | 0.00 | 0.270 | 0.073 | -0.184 | 0.000 |
| AR | 317042907 | 11 | 350350 | 0.00 | 0.275 | 0.071 | -0.179 | 0.000 |
| AR | 317048313 | 11 | 355756 | 0.00 | 0.278 | 0.074 | 0.189 | 0.078 |
| AR | 317050567 | 11 | 358010 | 0.00 | 0.285 | 0.069 | 0.176 | 0.026 |
| AR | 322908746 | 11 | 6216189 | 0.00 | 0.051 | 0.076 | -0.308 | 0.000 |
| AR | 323072783 | 11 | 6380226 | 0.00 | 0.104 | 0.094 | 0.279 | 0.000 |
| AR | 323086759 | 11 | 6394202 | 0.00 | 0.053 | 0.152 | -0.430 | 0.000 |
| AR | 324326504 | 11 | 7633947 | 0.00 | 0.104 | 0.100 | -0.259 | 0.000 |
| AR | 324345366 | 11 | 7652809 | 0.00 | 0.295 | 0.068 | 0.171 | 0.000 |
| AR | 326129582 | 11 | 9437025 | 0.00 | 0.076 | 0.080 | 0.260 | 0.000 |
| AR | 326135624 | 11 | 9443067 | 0.00 | 0.101 | 0.073 | 0.217 | 0.000 |
| AR | 355792284 | 12 | 10078621 | 0.00 | 0.096 | 0.093 | -0.438 | 0.398 |
| AR | 355948994 | 12 | 10235331 | 0.00 | 0.073 | 0.098 | 0.299 | 0.000 |
| AR | 357882334 | 12 | 12168671 | 0.00 | 0.136 | 0.071 | -0.214 | 0.000 |
| AR | 361473094 | 12 | 15759431 | 0.00 | 0.111 | 0.079 | -0.237 | 0.000 |
| AR | 361505371 | 12 | 15791708 | 0.00 | 0.088 | 0.070 | 0.219 | 0.000 |
| AR | 361505571 | 12 | 15791908 | 0.00 | 0.076 | 0.086 | 0.260 | 1.172 |
| AR | 361515087 | 12 | 15801424 | 0.00 | 0.056 | 0.094 | -0.329 | 0.098 |
| AR | 361519943 | 12 | 15806280 | 0.00 | 0.051 | 0.100 | -0.349 | 0.114 |
| AR | 361523188 | 12 | 15809525 | 0.00 | 0.051 | 0.100 | -0.349 | 0.087 |
| AR | 361528711 | 12 | 15815048 | 0.00 | 0.159 | 0.068 | 0.177 | 0.000 |
| AR | 361533333 | 12 | 15819670 | 0.00 | 0.061 | 0.119 | -0.352 | 0.040 |
| AR | 361568831 | 12 | 15855168 | 0.00 | 0.068 | 0.071 | 0.258 | 0.000 |
| AR | 361571592 | 12 | 15857929 | 0.00 | 0.063 | 0.080 | 0.286 | 2.073 |
| AR | 361619217 | 12 | 15905554 | 0.00 | 0.073 | 0.103 | -0.301 | 20.284 |
| AR | 361676445 | 12 | 15962782 | 0.00 | 0.237 | 0.067 | -0.144 | 0.000 |
| AR | 361682139 | 12 | 15968476 | 0.00 | 0.058 | 0.072 | 0.276 | 0.000 |
| AR | 361683376 | 12 | 15969713 | 0.00 | 0.091 | 0.070 | 0.232 | 0.000 |
| AR | 361701493 | 12 | 15987830 | 0.00 | 0.058 | 0.072 | 0.276 | 0.000 |
| AR | 361702764 | 12 | 15989101 | 0.00 | 0.242 | 0.074 | -0.156 | 0.021 |
| AR | 361706997 | 12 | 15993334 | 0.00 | 0.247 | 0.073 | 0.155 | 7.228 |
| AR | 361852496 | 12 | 16138833 | 0.00 | 0.063 | 0.088 | 0.299 | 0.028 |
| AR | 361854605 | 12 | 16140942 | 0.00 | 0.073 | 0.076 | 0.266 | 1.349 |
| AR | 361856895 | 12 | 16143232 | 0.00 | 0.071 | 0.076 | 0.262 | 0.004 |
| AR | 361860011 | 12 | 16146348 | 0.00 | 0.073 | 0.117 | 0.323 | 0.000 |
| AR | 361866090 | 12 | 16152427 | 0.00 | 0.081 | 0.106 | 0.293 | 2.166 |
| AR | 361866199 | 12 | 16152536 | 0.00 | 0.131 | 0.088 | 0.218 | 2.968 |
| AR | 361873419 | 12 | 16159756 | 0.00 | 0.071 | 0.166 | 0.394 | 0.000 |
| AR | 361874291 | 12 | 16160628 | 0.00 | 0.083 | 0.138 | 0.337 | 0.012 |
| AR | 361881387 | 12 | 16167724 | 0.00 | 0.154 | 0.074 | -0.212 | 0.000 |
| AR | 361883125 | 12 | 16169462 | 0.00 | 0.071 | 0.126 | 0.346 | 0.000 |
| AR | 361885832 | 12 | 16172169 | 0.00 | 0.071 | 0.166 | -0.394 | 0.055 |
| AR | 361890289 | 12 | 16176626 | 0.00 | 0.096 | 0.130 | 0.329 | 0.000 |
| AR | 361891074 | 12 | 16177411 | 0.00 | 0.144 | 0.091 | -0.278 | 0.000 |
| AR | 361906235 | 12 | 16192572 | 0.00 | 0.076 | 0.114 | -0.316 | 0.533 |
| AR | 361907239 | 12 | 16193576 | 0.00 | 0.134 | 0.104 | 0.315 | 0.000 |
| AR | 361909854 | 12 | 16196191 | 0.00 | 0.126 | 0.077 | -0.197 | 0.000 |
| AR | 361913418 | 12 | 16199755 | 0.00 | 0.078 | 0.148 | 0.355 | 0.000 |
| AR | 361917535 | 12 | 16203872 | 0.00 | 0.073 | 0.117 | 0.323 | 0.000 |
| AR | 361921986 | 12 | 16208323 | 0.00 | 0.071 | 0.083 | -0.269 | 0.000 |
| AR | 361969346 | 12 | 16255683 | 0.00 | 0.146 | 0.113 | -0.312 | 0.000 |
| AR | 361971036 | 12 | 16257373 | 0.00 | 0.124 | 0.075 | -0.225 | 0.000 |
| AR | 361971321 | 12 | 16257658 | 0.00 | 0.083 | 0.103 | 0.292 | 0.199 |
| AR | 361974885 | 12 | 16261222 | 0.00 | 0.096 | 0.082 | 0.243 | 0.253 |
| AR | 361974955 | 12 | 16261292 | 0.00 | 0.076 | 0.155 | 0.370 | 0.000 |
| AR | 361974958 | 12 | 16261295 | 0.00 | 0.134 | 0.075 | 0.192 | 0.000 |
| AR | 361989464 | 12 | 16275801 | 0.00 | 0.101 | 0.105 | -0.300 | 3.030 |
| AR | 361994182 | 12 | 16280519 | 0.00 | 0.144 | 0.068 | -0.181 | 0.000 |
| AR | 361996232 | 12 | 16282569 | 0.00 | 0.144 | 0.074 | -0.193 | 0.090 |
| AR | 361999293 | 12 | 16285630 | 0.00 | 0.129 | 0.081 | -0.208 | 0.136 |
| AR | 362001392 | 12 | 16287729 | 0.00 | 0.114 | 0.088 | 0.224 | 0.000 |
| AR | 362008564 | 12 | 16294901 | 0.00 | 0.063 | 0.088 | 0.299 | 0.064 |
| AR | 362024476 | 12 | 16310813 | 0.00 | 0.063 | 0.088 | 0.299 | 0.164 |
| AR | 362031019 | 12 | 16317356 | 0.00 | 0.071 | 0.075 | 0.260 | 0.000 |
| AR | 362111725 | 12 | 16398062 | 0.00 | 0.076 | 0.090 | -0.281 | 0.000 |
| AR | 362176760 | 12 | 16463097 | 0.00 | 0.210 | 0.076 | -0.207 | 0.614 |
| AR | 362799949 | 12 | 17086286 | 0.00 | 0.058 | 0.072 | 0.306 | 0.000 |
| AR | 362981416 | 12 | 17267753 | 0.00 | 0.096 | 0.070 | 0.357 | 0.052 |
| AR | 362995460 | 12 | 17281797 | 0.00 | 0.124 | 0.080 | 0.423 | 0.000 |
| AR | 363008820 | 12 | 17295157 | 0.00 | 0.076 | 0.068 | -0.251 | 0.000 |
| AR | 363013357 | 12 | 17299694 | 0.00 | 0.061 | 0.068 | -0.287 | 0.047 |
| AR | 363040828 | 12 | 17327165 | 0.00 | 0.076 | 0.087 | -0.300 | 0.000 |
| AR | 363046422 | 12 | 17332759 | 0.00 | 0.076 | 0.082 | 0.284 | 0.000 |
| AR | 363048823 | 12 | 17335160 | 0.00 | 0.061 | 0.068 | 0.287 | 0.000 |
| AR | 363052703 | 12 | 17339040 | 0.00 | 0.129 | 0.093 | 0.226 | 0.068 |
| AR | 363109724 | 12 | 17396061 | 0.00 | 0.056 | 0.077 | -0.313 | 0.000 |
| AR | 363119498 | 12 | 17405835 | 0.00 | 0.071 | 0.093 | -0.321 | 0.000 |
| AR | 363123691 | 12 | 17410028 | 0.00 | 0.053 | 0.072 | 0.317 | 0.018 |
| AR | 363127991 | 12 | 17414328 | 0.00 | 0.051 | 0.076 | 0.337 | 0.000 |
| AR | 363128351 | 12 | 17414688 | 0.00 | 0.063 | 0.090 | -0.322 | 0.000 |
| AR | 363137195 | 12 | 17423532 | 0.00 | 0.053 | 0.072 | -0.317 | 0.000 |
| AR | 363139495 | 12 | 17425832 | 0.00 | 0.073 | 0.088 | -0.311 | 0.000 |
| AR | 363139523 | 12 | 17425860 | 0.00 | 0.111 | 0.075 | -0.220 | 1.303 |
| AR | 363140218 | 12 | 17426555 | 0.00 | 0.056 | 0.072 | -0.321 | 0.000 |
| AR | 363140378 | 12 | 17426715 | 0.00 | 0.066 | 0.100 | -0.348 | 0.149 |
| AR | 363150724 | 12 | 17437061 | 0.00 | 0.131 | 0.129 | 0.537 | 0.000 |
| AR | 363153762 | 12 | 17440099 | 0.00 | 0.073 | 0.122 | -0.382 | 0.026 |
| AR | 363175817 | 12 | 17462154 | 0.00 | 0.053 | 0.072 | -0.317 | 0.000 |
| AR | 363180943 | 12 | 17467280 | 0.00 | 0.071 | 0.091 | -0.332 | 0.000 |
| AR | 363243959 | 12 | 17530296 | 0.00 | 0.053 | 0.072 | -0.303 | 0.000 |
| AR | 363639025 | 12 | 17925362 | 0.00 | 0.066 | 0.104 | -0.334 | 0.014 |
| AR | 363658921 | 12 | 17945258 | 0.00 | 0.061 | 0.077 | 0.322 | 0.000 |
| AR | 363663372 | 12 | 17949709 | 0.00 | 0.066 | 0.088 | 0.318 | 0.000 |
| AR | 366829025 | 12 | 21115362 | 0.00 | 0.152 | 0.072 | -0.182 | 0.000 |
| AR | 366834961 | 12 | 21121298 | 0.00 | 0.136 | 0.105 | 0.232 | 0.000 |
| AR | 366838282 | 12 | 21124619 | 0.00 | 0.101 | 0.111 | -0.273 | 0.000 |
| AR | 366838946 | 12 | 21125283 | 0.00 | 0.109 | 0.136 | 0.296 | 0.000 |
| AR | 366847803 | 12 | 21134140 | 0.00 | 0.101 | 0.073 | -0.216 | 0.232 |
| AR | 367488626 | 12 | 21774963 | 0.00 | 0.071 | 0.073 | -0.269 | 0.000 |
| AR | 367821503 | 12 | 22107840 | 0.00 | 0.328 | 0.072 | 0.153 | 0.012 |
| AR | 367822542 | 12 | 22108879 | 0.00 | 0.235 | 0.074 | 0.170 | 0.130 |
| AR | 367837531 | 12 | 22123868 | 0.00 | 0.230 | 0.075 | -0.173 | 0.000 |
| AR | 368044122 | 12 | 22330459 | 0.00 | 0.086 | 0.106 | 0.279 | 0.000 |
| AR | 368046648 | 12 | 22332985 | 0.00 | 0.081 | 0.081 | -0.247 | 0.063 |
| AR | 371302258 | 12 | 25588595 | 0.00 | 0.396 | 0.070 | 0.132 | 0.000 |
| AR | 372921475 | 12 | 27207812 | 0.00 | 0.139 | 0.077 | -0.288 | 0.000 |
| AR | 372926465 | 12 | 27212802 | 0.00 | 0.141 | 0.075 | 0.282 | 0.000 |
| AR | 372928649 | 12 | 27214986 | 0.00 | 0.139 | 0.075 | -0.279 | 0.000 |
| AR | 372932532 | 12 | 27218869 | 0.00 | 0.141 | 0.075 | -0.282 | 0.000 |
| AR | 372939013 | 12 | 27225350 | 0.00 | 0.141 | 0.075 | -0.282 | 0.801 |
| AR | 372943678 | 12 | 27230015 | 0.00 | 0.139 | 0.077 | 0.288 | 0.004 |
| AR | 372960998 | 12 | 27247335 | 0.00 | 0.141 | 0.075 | -0.282 | 3.079 |
| AR | 372967453 | 12 | 27253790 | 0.00 | 0.141 | 0.075 | -0.282 | 17.750 |
| AR | 372969532 | 12 | 27255869 | 0.00 | 0.141 | 0.075 | -0.282 | 3.087 |
| AR | 372976935 | 12 | 27263272 | 0.00 | 0.136 | 0.081 | -0.306 | 0.000 |
| AR | 372983686 | 12 | 27270023 | 0.00 | 0.134 | 0.081 | -0.304 | 0.000 |
| AR | 372990058 | 12 | 27276395 | 0.00 | 0.134 | 0.083 | 0.313 | 0.069 |
| AR | 350240107 | 12 | 4526444 | 0.00 | 0.068 | 0.087 | -0.293 | 0.000 |
| AR | 350263583 | 12 | 4549920 | 0.00 | 0.056 | 0.088 | 0.323 | 0.200 |
| AR | 350264559 | 12 | 4550896 | 0.00 | 0.066 | 0.071 | 0.263 | 0.804 |
| AR | 350267089 | 12 | 4553426 | 0.00 | 0.063 | 0.073 | 0.272 | 0.000 |
| AR | 350677750 | 12 | 4964087 | 0.00 | 0.056 | 0.077 | 0.291 | 0.000 |
| AR | 350748922 | 12 | 5035259 | 0.00 | 0.051 | 0.088 | 0.316 | 0.009 |
| AR | 351225965 | 12 | 5512302 | 0.00 | 0.053 | 0.082 | 0.317 | 2.114 |
| AR | 351239739 | 12 | 5526076 | 0.00 | 0.245 | 0.068 | -0.211 | 0.000 |
| AR | 351297872 | 12 | 5584209 | 0.00 | 0.061 | 0.069 | 0.268 | 0.056 |
| AR | 352673473 | 12 | 6959810 | 0.00 | 0.260 | 0.069 | 0.178 | 0.000 |
| AR | 353417261 | 12 | 7703598 | 0.00 | 0.144 | 0.081 | -0.279 | 0.000 |
| AR | 353422984 | 12 | 7709321 | 0.00 | 0.121 | 0.073 | 0.363 | 0.006 |
| AR | 353433310 | 12 | 7719647 | 0.00 | 0.063 | 0.069 | -0.357 | 0.000 |
| AR | 354129855 | 12 | 8416192 | 0.00 | 0.051 | 0.071 | 0.309 | 0.000 |
| HRR (%) | 13962048 | 1 | 13962048 | 0.00 | 0.472 | 0.058 | 3.728 | 0 |
| HRR (%) | 18610534 | 1 | 18610534 | 0.00 | 0.359 | 0.059 | 4.670 | 0.00 |
| HRR (%) | 19414776 | 1 | 19414776 | 0.00 | 0.465 | 0.070 | 4.380 | 0.00 |
| HRR (%) | 30513568 | 1 | 30513568 | 0.00 | 0.066 | 0.058 | 7.806 | 0.04 |
| HRR (%) | 34383222 | 1 | 34383222 | 0.00 | 0.104 | 0.068 | -10.369 | 0.00 |
| HRR (%) | 40816230 | 1 | 40816230 | 0.00 | 0.061 | 0.058 | 8.198 | 0.00 |
| HRR (%) | 42126513 | 1 | 42126513 | 0.00 | 0.086 | 0.059 | 8.094 | 0.02 |
| HRR (%) | 4267867 | 1 | 4267867 | 0.00 | 0.126 | 0.065 | 6.999 | 0.00 |
| HRR (%) | 4270117 | 1 | 4270117 | 0.00 | 0.126 | 0.065 | -6.999 | 0.01 |
| HRR (%) | 4291505 | 1 | 4291505 | 0.00 | 0.051 | 0.065 | -8.553 | 0.00 |
| HRR (%) | 4350753 | 1 | 4350753 | 0.00 | 0.078 | 0.069 | -7.340 | 0.00 |
| HRR (%) | 4353208 | 1 | 4353208 | 0.00 | 0.078 | 0.069 | 7.340 | 0.00 |
| HRR (%) | 4355517 | 1 | 4355517 | 0.00 | 0.078 | 0.069 | -7.340 | 0.00 |
| HRR (%) | 4369645 | 1 | 4369645 | 0.00 | 0.051 | 0.091 | -11.438 | 0.00 |
| HRR (%) | 4412090 | 1 | 4412090 | 0.00 | 0.341 | 0.058 | -4.395 | 0.00 |
| HRR (%) | 304607339 | 10 | 11122069 | 0.00 | 0.192 | 0.059 | -4.653 | 0.00 |
| HRR (%) | 309745917 | 10 | 16260647 | 0.00 | 0.152 | 0.062 | -6.644 | 0.00 |
| HRR (%) | 309788567 | 10 | 16303297 | 0.00 | 0.184 | 0.062 | 5.853 | 0.00 |
| HRR (%) | 309796894 | 10 | 16311624 | 0.00 | 0.152 | 0.062 | -6.644 | 0.01 |
| HRR (%) | 309801042 | 10 | 16315772 | 0.00 | 0.152 | 0.062 | -6.644 | 0.00 |
| HRR (%) | 300056232 | 10 | 6570962 | 0.00 | 0.285 | 0.061 | 4.843 | 0.00 |
| HRR (%) | 300368999 | 10 | 6883729 | 0.00 | 0.288 | 0.062 | 4.847 | 0.00 |
| HRR (%) | 300374705 | 10 | 6889435 | 0.00 | 0.283 | 0.058 | 4.813 | 0.00 |
| HRR (%) | 300406358 | 10 | 6921088 | 0.00 | 0.376 | 0.062 | -4.850 | 0.01 |
| HRR (%) | 301127577 | 10 | 7642307 | 0.00 | 0.288 | 0.065 | -4.969 | 0.00 |
| HRR (%) | 333605745 | 11 | 16913188 | 0.00 | 0.071 | 0.060 | -7.339 | 0.00 |
| HRR (%) | 336198804 | 11 | 19506247 | 0.00 | 0.116 | 0.060 | -5.642 | 0.00 |
| HRR (%) | 336254478 | 11 | 19561921 | 0.00 | 0.086 | 0.062 | 7.094 | 0.00 |
| HRR (%) | 336283140 | 11 | 19590583 | 0.00 | 0.192 | 0.072 | 5.108 | 0.00 |
| HRR (%) | 336313357 | 11 | 19620800 | 0.00 | 0.280 | 0.080 | -4.652 | 0.00 |
| HRR (%) | 336314352 | 11 | 19621795 | 0.00 | 0.245 | 0.066 | 4.444 | 0.00 |
| HRR (%) | 338315691 | 11 | 21623134 | 0.00 | 0.273 | 0.080 | 5.620 | 0.00 |
| HRR (%) | 341143743 | 11 | 24451186 | 0.00 | 0.495 | 0.062 | 4.329 | 0.00 |
| HRR (%) | 341145831 | 11 | 24453274 | 0.00 | 0.341 | 0.062 | 4.273 | 0.00 |
| HRR (%) | 341148868 | 11 | 24456311 | 0.00 | 0.056 | 0.080 | 9.599 | 0.00 |
| HRR (%) | 341354049 | 11 | 24661492 | 0.00 | 0.154 | 0.060 | -7.501 | 0.00 |
| HRR (%) | 341426481 | 11 | 24733924 | 0.00 | 0.053 | 0.059 | 8.147 | 0.00 |
| HRR (%) | 342314429 | 11 | 25621872 | 0.00 | 0.258 | 0.065 | 4.487 | 0.00 |
| HRR (%) | 342537927 | 11 | 25845370 | 0.00 | 0.184 | 0.060 | 4.707 | 0.00 |
| HRR (%) | 342540340 | 11 | 25847783 | 0.00 | 0.106 | 0.072 | -6.853 | 0.00 |
| HRR (%) | 342542512 | 11 | 25849955 | 0.00 | 0.109 | 0.082 | -7.284 | 0.24 |
| HRR (%) | 342543594 | 11 | 25851037 | 0.00 | 0.131 | 0.066 | 5.882 | 0.01 |
| HRR (%) | 342546854 | 11 | 25854297 | 0.00 | 0.106 | 0.072 | -6.853 | 0.00 |
| HRR (%) | 342574265 | 11 | 25881708 | 0.00 | 0.242 | 0.097 | 5.908 | 0.07 |
| HRR (%) | 342578311 | 11 | 25885754 | 0.00 | 0.167 | 0.076 | 5.566 | 0.00 |
| HRR (%) | 343023737 | 11 | 26331180 | 0.00 | 0.245 | 0.066 | -4.423 | 0.00 |
| HRR (%) | 343036125 | 11 | 26343568 | 0.00 | 0.192 | 0.067 | 5.882 | 0.00 |
| HRR (%) | 343075408 | 11 | 26382851 | 0.00 | 0.121 | 0.061 | -5.896 | 0.00 |
| HRR (%) | 343077560 | 11 | 26385003 | 0.00 | 0.159 | 0.064 | -5.350 | 0.00 |
| HRR (%) | 343079199 | 11 | 26386642 | 0.00 | 0.434 | 0.079 | -4.373 | 0.00 |
| HRR (%) | 343209799 | 11 | 26517242 | 0.00 | 0.139 | 0.068 | 5.690 | 0.00 |
| HRR (%) | 344688985 | 11 | 27996428 | 0.00 | 0.136 | 0.077 | 6.185 | 0.02 |
| HRR (%) | 344689554 | 11 | 27996997 | 0.00 | 0.199 | 0.082 | 5.496 | 0.00 |
| HRR (%) | 345549958 | 11 | 28857401 | 0.00 | 0.056 | 0.063 | 8.253 | 0.00 |
| HRR (%) | 345554936 | 11 | 28862379 | 0.00 | 0.061 | 0.060 | 7.794 | 1.03 |
| HRR (%) | 345558985 | 11 | 28866428 | 0.00 | 0.061 | 0.060 | 7.794 | 0.00 |
| HRR (%) | 345564147 | 11 | 28871590 | 0.00 | 0.061 | 0.060 | 7.794 | 0.00 |
| HRR (%) | 345571505 | 11 | 28878948 | 0.00 | 0.061 | 0.060 | 7.794 | 0.00 |
| HRR (%) | 345575887 | 11 | 28883330 | 0.00 | 0.061 | 0.060 | -7.794 | 0.00 |
| HRR (%) | 345577937 | 11 | 28885380 | 0.00 | 0.061 | 0.060 | 7.794 | 0.00 |
| HRR (%) | 345582710 | 11 | 28890153 | 0.00 | 0.061 | 0.060 | 7.794 | 0.00 |
| HRR (%) | 345588762 | 11 | 28896205 | 0.00 | 0.056 | 0.076 | -9.095 | 0.00 |
| HRR (%) | 345591783 | 11 | 28899226 | 0.00 | 0.063 | 0.088 | 9.346 | 0.00 |
| HRR (%) | 345597237 | 11 | 28904680 | 0.00 | 0.053 | 0.068 | -8.886 | 0.00 |
| HRR (%) | 345599519 | 11 | 28906962 | 0.00 | 0.056 | 0.076 | 9.095 | 0.00 |
| HRR (%) | 345603390 | 11 | 28910833 | 0.00 | 0.056 | 0.076 | -9.095 | 0.00 |
| HRR (%) | 345606137 | 11 | 28913580 | 0.00 | 0.053 | 0.078 | -9.528 | 0.00 |
| HRR (%) | 345609146 | 11 | 28916589 | 0.00 | 0.056 | 0.076 | 9.095 | 0.00 |
| HRR (%) | 345611287 | 11 | 28918730 | 0.00 | 0.066 | 0.076 | 8.336 | 0.00 |
| HRR (%) | 345635866 | 11 | 28943309 | 0.00 | 0.109 | 0.089 | 7.338 | 0.00 |
| HRR (%) | 345695170 | 11 | 29002613 | 0.00 | 0.051 | 0.058 | 8.297 | 0.00 |
| HRR (%) | 363052703 | 12 | 17339040 | 0.00 | 0.129 | 0.063 | 5.769 | 0.00 |
| HRR (%) | 365157574 | 12 | 19443911 | 0.00 | 0.470 | 0.061 | 3.970 | 0.00 |
| HRR (%) | 365165587 | 12 | 19451924 | 0.00 | 0.381 | 0.068 | 4.269 | 0.12 |
| HRR (%) | 365173802 | 12 | 19460139 | 0.00 | 0.495 | 0.058 | -3.910 | 0.00 |
| HRR (%) | 365173931 | 12 | 19460268 | 0.00 | 0.399 | 0.065 | 4.095 | 0.00 |
| HRR (%) | 365616636 | 12 | 19902973 | 0.00 | 0.366 | 0.066 | -4.042 | 0.00 |
| HRR (%) | 365627106 | 12 | 19913443 | 0.00 | 0.449 | 0.058 | -3.911 | 0.00 |
| HRR (%) | 366117970 | 12 | 20404307 | 0.00 | 0.434 | 0.060 | 3.820 | 0.00 |
| HRR (%) | 366444342 | 12 | 20730679 | 0.00 | 0.457 | 0.058 | -3.663 | 0.00 |
| HRR (%) | 367496253 | 12 | 21782590 | 0.00 | 0.066 | 0.061 | -7.888 | 0.01 |
| HRR (%) | 367624071 | 12 | 21910408 | 0.00 | 0.086 | 0.061 | 7.261 | 0.00 |
| HRR (%) | 367770858 | 12 | 22057195 | 0.00 | 0.104 | 0.065 | 7.272 | 0.00 |
| HRR (%) | 367803774 | 12 | 22090111 | 0.00 | 0.318 | 0.066 | -4.321 | 0.00 |
| HRR (%) | 354381901 | 12 | 8668238 | 0.00 | 0.220 | 0.058 | 4.968 | 0.00 |
| HRR (%) | 66987043 | 2 | 23716120 | 0.00 | 0.051 | 0.071 | 8.860 | 0.00 |
| HRR (%) | 68007443 | 2 | 24736520 | 0.00 | 0.477 | 0.064 | 3.903 | 0.00 |
| HRR (%) | 68023319 | 2 | 24752396 | 0.00 | 0.268 | 0.066 | 4.357 | 0.00 |
| HRR (%) | 68208941 | 2 | 24938018 | 0.00 | 0.354 | 0.065 | 4.088 | 0.00 |
| HRR (%) | 68347527 | 2 | 25076604 | 0.00 | 0.434 | 0.060 | 3.828 | 0.00 |
| HRR (%) | 68766269 | 2 | 25495346 | 0.00 | 0.268 | 0.071 | 4.514 | 0.00 |
| HRR (%) | 68888261 | 2 | 25617338 | 0.00 | 0.326 | 0.074 | -4.485 | 0.00 |
| HRR (%) | 68907025 | 2 | 25636102 | 0.00 | 0.351 | 0.060 | 4.213 | 0.00 |
| HRR (%) | 68907692 | 2 | 25636769 | 0.00 | 0.313 | 0.066 | 4.254 | 0.00 |
| HRR (%) | 69323175 | 2 | 26052252 | 0.00 | 0.240 | 0.058 | 4.233 | 0.00 |
| HRR (%) | 69817705 | 2 | 26546782 | 0.00 | 0.058 | 0.058 | 8.809 | 0.00 |
| HRR (%) | 73100519 | 2 | 29829596 | 0.00 | 0.184 | 0.065 | -4.972 | 0.00 |
| HRR (%) | 76944644 | 2 | 33673721 | 0.00 | 0.152 | 0.058 | -8.351 | 0.01 |
| HRR (%) | 48775898 | 2 | 5504975 | 0.00 | 0.346 | 0.066 | -4.330 | 0.00 |
| HRR (%) | 48834287 | 2 | 5563364 | 0.00 | 0.359 | 0.069 | 4.299 | 0.09 |
| HRR (%) | 48834825 | 2 | 5563902 | 0.00 | 0.457 | 0.064 | -4.128 | 0.00 |
| HRR (%) | 48836594 | 2 | 5565671 | 0.00 | 0.359 | 0.070 | 4.335 | 0.00 |
| HRR (%) | 48838877 | 2 | 5567954 | 0.00 | 0.354 | 0.076 | 4.512 | 0.00 |
| HRR (%) | 48840905 | 2 | 5569982 | 0.00 | 0.348 | 0.070 | -4.378 | 0.00 |
| HRR (%) | 48846992 | 2 | 5576069 | 0.00 | 0.381 | 0.070 | -4.402 | 0.00 |
| HRR (%) | 48849386 | 2 | 5578463 | 0.00 | 0.356 | 0.072 | 4.387 | 0.00 |
| HRR (%) | 48852650 | 2 | 5581727 | 0.00 | 0.346 | 0.073 | -4.510 | 0.00 |
| HRR (%) | 48852655 | 2 | 5581732 | 0.00 | 0.396 | 0.075 | 4.582 | 0.00 |
| HRR (%) | 48854909 | 2 | 5583986 | 0.00 | 0.399 | 0.068 | 4.356 | 0.00 |
| HRR (%) | 48855243 | 2 | 5584320 | 0.00 | 0.354 | 0.067 | 4.273 | 0.00 |
| HRR (%) | 48861855 | 2 | 5590932 | 0.00 | 0.394 | 0.078 | -4.652 | 0.00 |
| HRR (%) | 48863899 | 2 | 5592976 | 0.00 | 0.351 | 0.076 | -4.515 | 0.00 |
| HRR (%) | 48864070 | 2 | 5593147 | 0.00 | 0.394 | 0.079 | -4.698 | 0.00 |
| HRR (%) | 48865962 | 2 | 5595039 | 0.00 | 0.351 | 0.076 | 4.515 | 0.00 |
| HRR (%) | 48869333 | 2 | 5598410 | 0.00 | 0.351 | 0.076 | -4.515 | 0.00 |
| HRR (%) | 48872335 | 2 | 5601412 | 0.00 | 0.343 | 0.072 | 4.433 | 0.00 |
| HRR (%) | 48874806 | 2 | 5603883 | 0.00 | 0.346 | 0.079 | 4.627 | 0.00 |
| HRR (%) | 48877340 | 2 | 5606417 | 0.00 | 0.351 | 0.074 | 4.482 | 0.00 |
| HRR (%) | 48881471 | 2 | 5610548 | 0.00 | 0.351 | 0.069 | 4.306 | 0.00 |
| HRR (%) | 48887181 | 2 | 5616258 | 0.00 | 0.371 | 0.085 | 4.733 | 0.00 |
| HRR (%) | 48893314 | 2 | 5622391 | 0.00 | 0.364 | 0.084 | 4.765 | 0.00 |
| HRR (%) | 48895704 | 2 | 5624781 | 0.00 | 0.306 | 0.063 | -4.554 | 0.00 |
| HRR (%) | 44251805 | 2 | 980882 | 0.00 | 0.068 | 0.058 | -7.915 | 0.00 |
| HRR (%) | 97049161 | 3 | 17840988 | 0.00 | 0.063 | 0.101 | 11.144 | 0.57 |
| HRR (%) | 103033700 | 3 | 23825527 | 0.00 | 0.270 | 0.063 | -4.521 | 0.00 |
| HRR (%) | 107145485 | 3 | 27937312 | 0.00 | 0.096 | 0.074 | -11.030 | 0.00 |
| HRR (%) | 107676417 | 3 | 28468244 | 0.00 | 0.066 | 0.073 | 10.146 | 0.00 |
| HRR (%) | 108707219 | 3 | 29499046 | 0.00 | 0.066 | 0.060 | -9.296 | 0.00 |
| HRR (%) | 108743696 | 3 | 29535523 | 0.00 | 0.071 | 0.075 | 9.819 | 0.00 |
| HRR (%) | 108758575 | 3 | 29550402 | 0.00 | 0.066 | 0.060 | -9.296 | 0.00 |
| HRR (%) | 108788261 | 3 | 29580088 | 0.00 | 0.071 | 0.075 | -9.819 | 0.00 |
| HRR (%) | 108809564 | 3 | 29601391 | 0.00 | 0.071 | 0.075 | -9.819 | 0.00 |
| HRR (%) | 108820565 | 3 | 29612392 | 0.00 | 0.071 | 0.075 | 9.819 | 0.00 |
| HRR (%) | 108838196 | 3 | 29630023 | 0.00 | 0.071 | 0.075 | 9.819 | 0.41 |
| HRR (%) | 108850397 | 3 | 29642224 | 0.00 | 0.071 | 0.075 | -9.819 | 0.00 |
| HRR (%) | 108852637 | 3 | 29644464 | 0.00 | 0.056 | 0.067 | 10.766 | 0.03 |
| HRR (%) | 108883594 | 3 | 29675421 | 0.00 | 0.068 | 0.066 | -9.344 | 0.00 |
| HRR (%) | 108937033 | 3 | 29728860 | 0.00 | 0.066 | 0.080 | -10.769 | 0.00 |
| HRR (%) | 108941517 | 3 | 29733344 | 0.00 | 0.071 | 0.075 | 9.819 | 0.00 |
| HRR (%) | 109059646 | 3 | 29851473 | 0.00 | 0.071 | 0.075 | -9.819 | 0.00 |
| HRR (%) | 109090121 | 3 | 29881948 | 0.00 | 0.071 | 0.075 | -9.819 | 0.00 |
| HRR (%) | 109228602 | 3 | 30020429 | 0.00 | 0.061 | 0.069 | -10.057 | 0.01 |
| HRR (%) | 109240499 | 3 | 30032326 | 0.00 | 0.056 | 0.081 | -11.506 | 0.00 |
| HRR (%) | 109258858 | 3 | 30050685 | 0.00 | 0.058 | 0.066 | -10.133 | 0.00 |
| HRR (%) | 109285552 | 3 | 30077379 | 0.00 | 0.061 | 0.069 | -10.057 | 0.00 |
| HRR (%) | 109332079 | 3 | 30123906 | 0.00 | 0.058 | 0.066 | 10.133 | 0.00 |
| HRR (%) | 109449798 | 3 | 30241625 | 0.00 | 0.061 | 0.069 | -10.057 | 0.00 |
| HRR (%) | 109520729 | 3 | 30312556 | 0.00 | 0.061 | 0.069 | 10.057 | 0.00 |
| HRR (%) | 109621642 | 3 | 30413469 | 0.00 | 0.061 | 0.069 | -10.057 | 0.04 |
| HRR (%) | 109655402 | 3 | 30447229 | 0.00 | 0.061 | 0.069 | -10.057 | 0.00 |
| HRR (%) | 113045853 | 3 | 33837680 | 0.00 | 0.301 | 0.058 | 4.371 | 0.01 |
| HRR (%) | 86476340 | 3 | 7268167 | 0.00 | 0.222 | 0.059 | 4.447 | 0.04 |
| HRR (%) | 86529175 | 3 | 7321002 | 0.00 | 0.220 | 0.062 | -4.568 | 0.00 |
| HRR (%) | 87057845 | 3 | 7849672 | 0.00 | 0.356 | 0.059 | -4.293 | 0.00 |
| HRR (%) | 87443938 | 3 | 8235765 | 0.00 | 0.205 | 0.071 | -5.090 | 0.09 |
| HRR (%) | 87473479 | 3 | 8265306 | 0.00 | 0.210 | 0.067 | 4.912 | 0.00 |
| HRR (%) | 87532112 | 3 | 8323939 | 0.00 | 0.210 | 0.067 | 4.912 | 0.00 |
| HRR (%) | 87540123 | 3 | 8331950 | 0.00 | 0.210 | 0.067 | -4.912 | 0.00 |
| HRR (%) | 87553750 | 3 | 8345577 | 0.00 | 0.210 | 0.067 | -4.912 | 0.00 |
| HRR (%) | 87563032 | 3 | 8354859 | 0.00 | 0.210 | 0.067 | 4.912 | 0.00 |
| HRR (%) | 87566775 | 3 | 8358602 | 0.00 | 0.207 | 0.069 | -5.089 | 0.00 |
| HRR (%) | 87577396 | 3 | 8369223 | 0.00 | 0.215 | 0.063 | 4.742 | 0.01 |
| HRR (%) | 87644259 | 3 | 8436086 | 0.00 | 0.182 | 0.059 | 4.861 | 0.00 |
| HRR (%) | 87667065 | 3 | 8458892 | 0.00 | 0.184 | 0.063 | 4.954 | 0.00 |
| HRR (%) | 87694090 | 3 | 8485917 | 0.00 | 0.184 | 0.063 | 4.954 | 0.00 |
| HRR (%) | 87697311 | 3 | 8489138 | 0.00 | 0.184 | 0.063 | 4.954 | 0.07 |
| HRR (%) | 87705242 | 3 | 8497069 | 0.00 | 0.182 | 0.063 | -5.007 | 0.00 |
| HRR (%) | 87718145 | 3 | 8509972 | 0.00 | 0.179 | 0.059 | 4.909 | 0.41 |
| HRR (%) | 87751511 | 3 | 8543338 | 0.00 | 0.265 | 0.060 | 4.496 | 1.06 |
| HRR (%) | 87754117 | 3 | 8545944 | 0.00 | 0.263 | 0.064 | -4.679 | 0.00 |
| HRR (%) | 87756718 | 3 | 8548545 | 0.00 | 0.265 | 0.060 | 4.496 | 0.00 |
| HRR (%) | 87760277 | 3 | 8552104 | 0.00 | 0.265 | 0.060 | 4.496 | 0.00 |
| HRR (%) | 87766883 | 3 | 8558710 | 0.00 | 0.265 | 0.060 | 4.496 | 0.50 |
| HRR (%) | 87775330 | 3 | 8567157 | 0.00 | 0.265 | 0.060 | 4.496 | 0.00 |
| HRR (%) | 87789608 | 3 | 8581435 | 0.00 | 0.164 | 0.060 | -5.135 | 0.00 |
| HRR (%) | 138422230 | 4 | 22800238 | 0.00 | 0.442 | 0.058 | -3.680 | 0.02 |
| HRR (%) | 146738055 | 4 | 31116063 | 0.00 | 0.222 | 0.058 | -4.314 | 0.00 |
| HRR (%) | 146738216 | 4 | 31116224 | 0.00 | 0.351 | 0.080 | 5.085 | 0.00 |
| HRR (%) | 146789179 | 4 | 31167187 | 0.00 | 0.414 | 0.082 | -4.243 | 0.00 |
| HRR (%) | 146795485 | 4 | 31173493 | 0.00 | 0.407 | 0.061 | 3.673 | 0.00 |
| HRR (%) | 146797756 | 4 | 31175764 | 0.00 | 0.285 | 0.066 | -4.247 | 0.00 |
| HRR (%) | 146847512 | 4 | 31225520 | 0.00 | 0.439 | 0.059 | 3.721 | 0.00 |
| HRR (%) | 146855009 | 4 | 31233017 | 0.00 | 0.356 | 0.068 | 3.996 | 0.00 |
| HRR (%) | 146857063 | 4 | 31235071 | 0.00 | 0.422 | 0.061 | 3.693 | 0.11 |
| HRR (%) | 146860533 | 4 | 31238541 | 0.00 | 0.336 | 0.062 | -3.951 | 0.19 |
| HRR (%) | 198195676 | 6 | 17112556 | 0.00 | 0.119 | 0.072 | -7.539 | 0.00 |
| HRR (%) | 198210814 | 6 | 17127694 | 0.00 | 0.101 | 0.064 | 8.100 | 0.10 |
| HRR (%) | 209236491 | 6 | 28153371 | 0.00 | 0.068 | 0.063 | 8.837 | 0.00 |
| HRR (%) | 209255692 | 6 | 28172572 | 0.00 | 0.083 | 0.060 | 7.537 | 0.00 |
| HRR (%) | 184750602 | 6 | 3667482 | 0.00 | 0.106 | 0.108 | -9.179 | 0.00 |
| HRR (%) | 184795646 | 6 | 3712526 | 0.00 | 0.124 | 0.099 | 8.175 | 0.04 |
| HRR (%) | 184813165 | 6 | 3730045 | 0.00 | 0.141 | 0.109 | 7.869 | 0.00 |
| HRR (%) | 184836993 | 6 | 3753873 | 0.00 | 0.139 | 0.105 | -7.803 | 0.00 |
| HRR (%) | 184859992 | 6 | 3776872 | 0.00 | 0.066 | 0.068 | 9.618 | 0.00 |
| HRR (%) | 232745654 | 7 | 20413747 | 0.00 | 0.205 | 0.059 | -5.300 | 0.00 |
| HRR (%) | 239100982 | 7 | 26769075 | 0.00 | 0.318 | 0.069 | 5.153 | 0.36 |
| HRR (%) | 239103579 | 7 | 26771672 | 0.00 | 0.260 | 0.077 | 5.432 | 0.37 |
| HRR (%) | 239106090 | 7 | 26774183 | 0.00 | 0.326 | 0.060 | -4.305 | 0.00 |
| PGC (%) | 14464015 | 1 | 14464015 | 0.001 | 0.066 | 0.098 | -11.359 | 0.00 |
| PGC (%) | 14467727 | 1 | 14467727 | 0.001 | 0.058 | 0.096 | 11.989 | 0.00 |
| PGC (%) | 14474816 | 1 | 14474816 | 0.000 | 0.061 | 0.109 | 12.864 | 0.00 |
| PGC (%) | 14478999 | 1 | 14478999 | 0.000 | 0.061 | 0.109 | 12.864 | 0.05 |
| PGC (%) | 14479845 | 1 | 14479845 | 0.001 | 0.301 | 0.093 | -6.440 | 0.00 |
| PGC (%) | 14480369 | 1 | 14480369 | 0.000 | 0.061 | 0.109 | -12.864 | 0.00 |
| PGC (%) | 14481925 | 1 | 14481925 | 0.000 | 0.086 | 0.101 | -10.362 | 0.29 |
| PGC (%) | 28555369 | 1 | 28555369 | 0.000 | 0.073 | 0.102 | -11.069 | 0.00 |
| PGC (%) | 28564987 | 1 | 28564987 | 0.001 | 0.056 | 0.095 | -11.778 | 0.00 |
| PGC (%) | 28600292 | 1 | 28600292 | 0.001 | 0.056 | 0.095 | 11.778 | 0.00 |
| PGC (%) | 28603299 | 1 | 28603299 | 0.001 | 0.056 | 0.095 | -11.778 | 0.00 |
| PGC (%) | 28615065 | 1 | 28615065 | 0.001 | 0.056 | 0.095 | -11.778 | 0.00 |
| PGC (%) | 28650046 | 1 | 28650046 | 0.000 | 0.096 | 0.112 | -10.793 | 0.00 |
| PGC (%) | 40108822 | 1 | 40108822 | 0.001 | 0.253 | 0.096 | -8.094 | 0.00 |
| PGC (%) | 307995519 | 10 | 14510249 | 0.000 | 0.255 | 0.105 | -6.898 | 0.00 |
| PGC (%) | 307995930 | 10 | 14510660 | 0.001 | 0.220 | 0.096 | 6.812 | 0.00 |
| PGC (%) | 307997749 | 10 | 14512479 | 0.000 | 0.217 | 0.100 | -7.103 | 0.00 |
| PGC (%) | 307998713 | 10 | 14513443 | 0.001 | 0.389 | 0.092 | -6.105 | 0.00 |
| PGC (%) | 308002797 | 10 | 14517527 | 0.000 | 0.207 | 0.103 | -7.332 | 0.00 |
| PGC (%) | 308003201 | 10 | 14517931 | 0.001 | 0.386 | 0.095 | 6.223 | 0.00 |
| PGC (%) | 308005395 | 10 | 14520125 | 0.000 | 0.225 | 0.099 | -6.734 | 0.00 |
| PGC (%) | 308006255 | 10 | 14520985 | 0.001 | 0.389 | 0.094 | 6.193 | 0.00 |
| PGC (%) | 308008689 | 10 | 14523419 | 0.000 | 0.394 | 0.100 | 6.528 | 0.00 |
| PGC (%) | 308009970 | 10 | 14524700 | 0.000 | 0.207 | 0.103 | -7.317 | 0.00 |
| PGC (%) | 308010016 | 10 | 14524746 | 0.001 | 0.374 | 0.096 | 6.428 | 0.02 |
| PGC (%) | 308017288 | 10 | 14532018 | 0.001 | 0.225 | 0.097 | -6.689 | 0.00 |
| PGC (%) | 308017824 | 10 | 14532554 | 0.001 | 0.386 | 0.095 | 6.223 | 0.04 |
| PGC (%) | 308034093 | 10 | 14548823 | 0.000 | 0.394 | 0.099 | -6.406 | 0.00 |
| PGC (%) | 308251538 | 10 | 14766268 | 0.001 | 0.101 | 0.094 | -9.169 | 0.00 |
| PGC (%) | 308311979 | 10 | 14826709 | 0.001 | 0.364 | 0.094 | -6.492 | 0.01 |
| PGC (%) | 308314908 | 10 | 14829638 | 0.001 | 0.366 | 0.094 | 6.499 | 0.00 |
| PGC (%) | 308329546 | 10 | 14844276 | 0.001 | 0.359 | 0.093 | 6.483 | 0.02 |
| PGC (%) | 295961412 | 10 | 2476142 | 0.001 | 0.253 | 0.096 | 8.506 | 0.05 |
| PGC (%) | 299573478 | 10 | 6088208 | 0.001 | 0.078 | 0.095 | -10.545 | 0.00 |
| PGC (%) | 331726331 | 11 | 15033774 | 0.001 | 0.293 | 0.095 | 7.269 | 0.00 |
| PGC (%) | 332021457 | 11 | 15328900 | 0.001 | 0.235 | 0.097 | -7.489 | 0.00 |
| PGC (%) | 332034701 | 11 | 15342144 | 0.001 | 0.275 | 0.098 | 7.030 | 0.40 |
| PGC (%) | 332476062 | 11 | 15783505 | 0.001 | 0.318 | 0.095 | -6.314 | 0.47 |
| PGC (%) | 332925685 | 11 | 16233128 | 0.001 | 0.482 | 0.094 | 6.348 | 0.00 |
| PGC (%) | 332935391 | 11 | 16242834 | 0.001 | 0.290 | 0.093 | 6.274 | 0.00 |
| PGC (%) | 333260269 | 11 | 16567712 | 0.000 | 0.063 | 0.103 | -14.089 | 0.00 |
| PGC (%) | 333270677 | 11 | 16578120 | 0.000 | 0.058 | 0.128 | -17.911 | 0.00 |
| PGC (%) | 333717157 | 11 | 17024600 | 0.000 | 0.351 | 0.103 | -6.807 | 0.00 |
| PGC (%) | 333754557 | 11 | 17062000 | 0.000 | 0.316 | 0.101 | 7.448 | 0.00 |
| PGC (%) | 333756343 | 11 | 17063786 | 0.000 | 0.414 | 0.102 | 6.595 | 0.14 |
| PGC (%) | 333756558 | 11 | 17064001 | 0.000 | 0.321 | 0.107 | -7.725 | 0.00 |
| PGC (%) | 333760112 | 11 | 17067555 | 0.000 | 0.359 | 0.128 | 8.260 | 0.05 |
| PGC (%) | 333760331 | 11 | 17067774 | 0.000 | 0.439 | 0.128 | 7.551 | 0.00 |
| PGC (%) | 333763494 | 11 | 17070937 | 0.000 | 0.331 | 0.120 | -8.385 | 0.00 |
| PGC (%) | 333765804 | 11 | 17073247 | 0.000 | 0.419 | 0.114 | 7.090 | 0.00 |
| PGC (%) | 333767030 | 11 | 17074473 | 0.000 | 0.328 | 0.121 | 8.516 | 0.00 |
| PGC (%) | 333813658 | 11 | 17121101 | 0.001 | 0.412 | 0.097 | 6.215 | 0.00 |
| PGC (%) | 333840638 | 11 | 17148081 | 0.001 | 0.134 | 0.092 | -8.210 | 0.00 |
| PGC (%) | 333859080 | 11 | 17166523 | 0.001 | 0.412 | 0.095 | -6.192 | 0.00 |
| PGC (%) | 333859742 | 11 | 17167185 | 0.000 | 0.157 | 0.107 | -8.624 | 0.00 |
| PGC (%) | 333868451 | 11 | 17175894 | 0.001 | 0.121 | 0.097 | 8.979 | 0.00 |
| PGC (%) | 333870076 | 11 | 17177519 | 0.000 | 0.417 | 0.103 | -7.120 | 0.02 |
| PGC (%) | 333871256 | 11 | 17178699 | 0.000 | 0.129 | 0.100 | -8.846 | 0.00 |
| PGC (%) | 333871600 | 11 | 17179043 | 0.000 | 0.364 | 0.127 | 7.591 | 0.00 |
| PGC (%) | 333874370 | 11 | 17181813 | 0.000 | 0.326 | 0.139 | -8.353 | 0.00 |
| PGC (%) | 333891845 | 11 | 17199288 | 0.000 | 0.129 | 0.100 | 8.846 | 0.00 |
| PGC (%) | 333895764 | 11 | 17203207 | 0.000 | 0.129 | 0.100 | 8.846 | 0.00 |
| PGC (%) | 333898065 | 11 | 17205508 | 0.000 | 0.129 | 0.100 | -8.846 | 0.00 |
| PGC (%) | 333929764 | 11 | 17237207 | 0.000 | 0.331 | 0.122 | -8.399 | 0.00 |
| PGC (%) | 334056689 | 11 | 17364132 | 0.000 | 0.273 | 0.101 | -6.857 | 0.00 |
| PGC (%) | 334059073 | 11 | 17366516 | 0.001 | 0.242 | 0.094 | 6.908 | 0.00 |
| PGC (%) | 334157571 | 11 | 17465014 | 0.000 | 0.210 | 0.099 | 7.223 | 0.00 |
| PGC (%) | 334258365 | 11 | 17565808 | 0.000 | 0.348 | 0.114 | -7.939 | 0.03 |
| PGC (%) | 334259197 | 11 | 17566640 | 0.001 | 0.414 | 0.094 | -5.831 | 0.00 |
| PGC (%) | 334260019 | 11 | 17567462 | 0.001 | 0.237 | 0.092 | 6.535 | 0.00 |
| PGC (%) | 334277053 | 11 | 17584496 | 0.001 | 0.247 | 0.094 | -6.830 | 0.00 |
| PGC (%) | 334288628 | 11 | 17596071 | 0.000 | 0.270 | 0.099 | -6.533 | 0.00 |
| PGC (%) | 340852414 | 11 | 24159857 | 0.001 | 0.061 | 0.097 | 11.436 | 0.02 |
| PGC (%) | 345123280 | 11 | 28430723 | 0.000 | 0.076 | 0.102 | -11.313 | 0.00 |
| PGC (%) | 345442853 | 11 | 28750296 | 0.001 | 0.073 | 0.097 | -10.868 | 0.00 |
| PGC (%) | 345454552 | 11 | 28761995 | 0.000 | 0.109 | 0.103 | -9.349 | 0.07 |
| PGC (%) | 345488557 | 11 | 28796000 | 0.000 | 0.131 | 0.103 | -8.715 | 0.00 |
| PGC (%) | 317536338 | 11 | 843781 | 0.000 | 0.081 | 0.120 | -12.239 | 0.00 |
| PGC (%) | 325209955 | 11 | 8517398 | 0.000 | 0.091 | 0.102 | -11.246 | 0.00 |
| PGC (%) | 363057393 | 12 | 17343730 | 0.001 | 0.063 | 0.093 | 11.036 | 0.05 |
| PGC (%) | 363080501 | 12 | 17366838 | 0.000 | 0.083 | 0.110 | 11.184 | 0.00 |
| PGC (%) | 363107185 | 12 | 17393522 | 0.001 | 0.071 | 0.098 | -11.032 | 0.00 |
| PGC (%) | 363571135 | 12 | 17857472 | 0.000 | 0.058 | 0.114 | 14.998 | 0.00 |
| PGC (%) | 363594430 | 12 | 17880767 | 0.000 | 0.063 | 0.135 | -15.628 | 0.00 |
| PGC (%) | 363634381 | 12 | 17920718 | 0.001 | 0.076 | 0.094 | -10.532 | 0.00 |
| PGC (%) | 363640531 | 12 | 17926868 | 0.000 | 0.073 | 0.103 | -11.138 | 0.00 |
| PGC (%) | 365384402 | 12 | 19670739 | 0.001 | 0.101 | 0.095 | 9.262 | 0.00 |
| PGC (%) | 365387548 | 12 | 19673885 | 0.001 | 0.101 | 0.095 | 9.262 | 0.00 |
| PGC (%) | 365389244 | 12 | 19675581 | 0.001 | 0.101 | 0.095 | 9.262 | 0.00 |
| PGC (%) | 365390000 | 12 | 19676337 | 0.001 | 0.101 | 0.095 | 9.262 | 0.00 |
| PGC (%) | 365390521 | 12 | 19676858 | 0.000 | 0.073 | 0.107 | -11.895 | 0.00 |
| PGC (%) | 365396664 | 12 | 19683001 | 0.000 | 0.182 | 0.101 | 7.866 | 0.00 |
| PGC (%) | 365396780 | 12 | 19683117 | 0.001 | 0.119 | 0.094 | -8.884 | 0.00 |
| PGC (%) | 365397402 | 12 | 19683739 | 0.000 | 0.111 | 0.099 | 9.288 | 0.00 |
| PGC (%) | 365402826 | 12 | 19689163 | 0.000 | 0.139 | 0.105 | 9.255 | 0.00 |
| PGC (%) | 365407580 | 12 | 19693917 | 0.000 | 0.104 | 0.099 | 9.875 | 0.00 |
| PGC (%) | 365407698 | 12 | 19694035 | 0.001 | 0.081 | 0.099 | 10.669 | 0.37 |
| PGC (%) | 365408299 | 12 | 19694636 | 0.000 | 0.111 | 0.099 | -9.288 | 0.02 |
| PGC (%) | 365413930 | 12 | 19700267 | 0.000 | 0.111 | 0.099 | 9.288 | 0.00 |
| PGC (%) | 365416645 | 12 | 19702982 | 0.001 | 0.139 | 0.098 | -8.829 | 0.00 |
| PGC (%) | 365419932 | 12 | 19706269 | 0.001 | 0.172 | 0.093 | -8.518 | 0.01 |
| PGC (%) | 365424194 | 12 | 19710531 | 0.000 | 0.172 | 0.100 | 7.901 | 0.00 |
| PGC (%) | 365425013 | 12 | 19711350 | 0.000 | 0.086 | 0.104 | 10.713 | 0.00 |
| PGC (%) | 365426111 | 12 | 19712448 | 0.001 | 0.116 | 0.096 | 8.875 | 0.00 |
| PGC (%) | 365428091 | 12 | 19714428 | 0.001 | 0.260 | 0.097 | -7.972 | 0.00 |
| PGC (%) | 365428440 | 12 | 19714777 | 0.000 | 0.111 | 0.099 | -9.288 | 1.44 |
| PGC (%) | 365432880 | 12 | 19719217 | 0.000 | 0.111 | 0.099 | -9.288 | 2.10 |
| PGC (%) | 365438936 | 12 | 19725273 | 0.000 | 0.111 | 0.099 | 9.288 | 0.00 |
| PGC (%) | 365443144 | 12 | 19729481 | 0.000 | 0.111 | 0.099 | 9.288 | 0.00 |
| PGC (%) | 365492678 | 12 | 19779015 | 0.001 | 0.083 | 0.092 | -10.062 | 0.04 |
| PGC (%) | 348799363 | 12 | 3085700 | 0.001 | 0.217 | 0.092 | -6.531 | 0.00 |
| PGC (%) | 348816860 | 12 | 3103197 | 0.000 | 0.172 | 0.128 | 9.326 | 0.00 |
| PGC (%) | 348821124 | 12 | 3107461 | 0.000 | 0.169 | 0.129 | -9.445 | 0.04 |
| PGC (%) | 348826855 | 12 | 3113192 | 0.000 | 0.167 | 0.135 | -9.731 | 0.00 |
| PGC (%) | 348829583 | 12 | 3115920 | 0.000 | 0.116 | 0.112 | 11.643 | 0.00 |
| PGC (%) | 348838949 | 12 | 3125286 | 0.000 | 0.184 | 0.141 | -9.663 | 0.00 |
| PGC (%) | 348842716 | 12 | 3129053 | 0.001 | 0.154 | 0.096 | 7.809 | 0.00 |
| PGC (%) | 348846237 | 12 | 3132574 | 0.001 | 0.152 | 0.098 | 8.056 | 0.00 |
| PGC (%) | 348854766 | 12 | 3141103 | 0.000 | 0.189 | 0.116 | -8.261 | 0.08 |
| PGC (%) | 348860789 | 12 | 3147126 | 0.000 | 0.187 | 0.107 | -7.853 | 0.12 |
| PGC (%) | 348870706 | 12 | 3157043 | 0.000 | 0.187 | 0.112 | 8.123 | 0.29 |
| PGC (%) | 348876599 | 12 | 3162936 | 0.000 | 0.162 | 0.104 | -8.485 | 0.00 |
| PGC (%) | 348882245 | 12 | 3168582 | 0.000 | 0.184 | 0.111 | -8.133 | 0.00 |
| PGC (%) | 54472421 | 2 | 11201498 | 0.001 | 0.063 | 0.098 | 11.920 | 0.02 |
| PGC (%) | 54688820 | 2 | 11417897 | 0.000 | 0.066 | 0.105 | 12.420 | 0.00 |
| PGC (%) | 54737512 | 2 | 11466589 | 0.001 | 0.071 | 0.093 | -11.137 | 0.00 |
| PGC (%) | 57772907 | 2 | 14501984 | 0.001 | 0.119 | 0.092 | -10.006 | 0.00 |
| PGC (%) | 61355273 | 2 | 18084350 | 0.001 | 0.056 | 0.095 | -12.633 | 0.00 |
| PGC (%) | 62924783 | 2 | 19653860 | 0.001 | 0.076 | 0.096 | -10.730 | 0.00 |
| PGC (%) | 63924049 | 2 | 20653126 | 0.000 | 0.053 | 0.115 | -14.143 | 0.00 |
| PGC (%) | 63940064 | 2 | 20669141 | 0.000 | 0.053 | 0.115 | -14.143 | 0.00 |
| PGC (%) | 64166076 | 2 | 20895153 | 0.001 | 0.308 | 0.099 | -6.645 | 0.00 |
| PGC (%) | 64168149 | 2 | 20897226 | 0.000 | 0.306 | 0.102 | 6.856 | 0.00 |
| PGC (%) | 64170518 | 2 | 20899595 | 0.001 | 0.308 | 0.099 | 6.645 | 0.00 |
| PGC (%) | 64172963 | 2 | 20902040 | 0.000 | 0.306 | 0.101 | -6.812 | 0.00 |
| PGC (%) | 64176357 | 2 | 20905434 | 0.001 | 0.308 | 0.099 | -6.645 | 0.02 |
| PGC (%) | 64180091 | 2 | 20909168 | 0.000 | 0.306 | 0.101 | -6.813 | 0.00 |
| PGC (%) | 64184541 | 2 | 20913618 | 0.001 | 0.328 | 0.092 | -6.092 | 0.00 |
| PGC (%) | 64188701 | 2 | 20917778 | 0.001 | 0.331 | 0.093 | -6.135 | 0.00 |
| PGC (%) | 64191876 | 2 | 20920953 | 0.000 | 0.346 | 0.105 | -6.601 | 0.00 |
| PGC (%) | 64195992 | 2 | 20925069 | 0.001 | 0.354 | 0.098 | 6.148 | 0.00 |
| PGC (%) | 64200893 | 2 | 20929970 | 0.000 | 0.351 | 0.100 | 6.246 | 0.00 |
| PGC (%) | 64299398 | 2 | 21028475 | 0.000 | 0.242 | 0.100 | -6.978 | 0.00 |
| PGC (%) | 65156286 | 2 | 21885363 | 0.001 | 0.063 | 0.098 | -12.143 | 0.00 |
| PGC (%) | 65195049 | 2 | 21924126 | 0.001 | 0.104 | 0.095 | 9.427 | 0.00 |
| PGC (%) | 65200257 | 2 | 21929334 | 0.001 | 0.091 | 0.097 | -10.611 | 0.00 |
| PGC (%) | 65210849 | 2 | 21939926 | 0.001 | 0.071 | 0.095 | -11.170 | 0.00 |
| PGC (%) | 65218973 | 2 | 21948050 | 0.001 | 0.071 | 0.095 | -11.170 | 0.00 |
| PGC (%) | 67493628 | 2 | 24222705 | 0.001 | 0.056 | 0.098 | -12.412 | 0.00 |
| PGC (%) | 67542265 | 2 | 24271342 | 0.001 | 0.126 | 0.094 | 8.550 | 0.03 |
| PGC (%) | 68224077 | 2 | 24953154 | 0.000 | 0.136 | 0.109 | 9.290 | 0.00 |
| PGC (%) | 68307390 | 2 | 25036467 | 0.000 | 0.167 | 0.107 | -8.311 | 0.01 |
| PGC (%) | 68321963 | 2 | 25051040 | 0.000 | 0.179 | 0.111 | 8.350 | 0.00 |
| PGC (%) | 68352105 | 2 | 25081182 | 0.000 | 0.134 | 0.129 | -11.657 | 0.00 |
| PGC (%) | 76858611 | 2 | 33587688 | 0.001 | 0.073 | 0.095 | -10.452 | 0.00 |
| PGC (%) | 77898973 | 2 | 34628050 | 0.000 | 0.139 | 0.102 | 9.618 | 0.00 |
| PGC (%) | 77908078 | 2 | 34637155 | 0.000 | 0.187 | 0.127 | -12.261 | 0.00 |
| PGC (%) | 77910183 | 2 | 34639260 | 0.000 | 0.184 | 0.118 | -11.891 | 0.00 |
| PGC (%) | 77915436 | 2 | 34644513 | 0.000 | 0.162 | 0.113 | -11.756 | 0.11 |
| PGC (%) | 77923106 | 2 | 34652183 | 0.000 | 0.495 | 0.129 | -7.904 | 0.00 |
| PGC (%) | 77925640 | 2 | 34654717 | 0.000 | 0.343 | 0.125 | -8.256 | 0.00 |
| PGC (%) | 77930082 | 2 | 34659159 | 0.000 | 0.351 | 0.113 | -7.604 | 0.03 |
| PGC (%) | 77936138 | 2 | 34665215 | 0.001 | 0.199 | 0.097 | -9.761 | 0.00 |
| PGC (%) | 78419700 | 2 | 35148777 | 0.000 | 0.157 | 0.100 | 8.426 | 0.03 |
| PGC (%) | 78422655 | 2 | 35151732 | 0.000 | 0.157 | 0.100 | 8.426 | 0.00 |
| PGC (%) | 78425581 | 2 | 35154658 | 0.000 | 0.199 | 0.106 | -7.862 | 0.00 |
| PGC (%) | 78426341 | 2 | 35155418 | 0.001 | 0.144 | 0.096 | 8.673 | 0.00 |
| PGC (%) | 78427820 | 2 | 35156897 | 0.000 | 0.197 | 0.100 | -7.588 | 0.00 |
| PGC (%) | 78429637 | 2 | 35158714 | 0.000 | 0.157 | 0.100 | -8.426 | 0.00 |
| PGC (%) | 78433392 | 2 | 35162469 | 0.000 | 0.162 | 0.123 | 9.739 | 0.00 |
| PGC (%) | 78437213 | 2 | 35166290 | 0.000 | 0.162 | 0.123 | -9.739 | 0.09 |
| PGC (%) | 78440220 | 2 | 35169297 | 0.000 | 0.205 | 0.106 | 7.991 | 0.00 |
| PGC (%) | 78445257 | 2 | 35174334 | 0.000 | 0.194 | 0.116 | 8.580 | 0.00 |
| PGC (%) | 50225136 | 2 | 6954213 | 0.001 | 0.071 | 0.093 | -10.783 | 0.00 |
| PGC (%) | 50779339 | 2 | 7508416 | 0.000 | 0.056 | 0.112 | -13.681 | 0.00 |
| PGC (%) | 50781811 | 2 | 7510888 | 0.000 | 0.061 | 0.116 | -13.712 | 0.00 |
| PGC (%) | 50786330 | 2 | 7515407 | 0.001 | 0.056 | 0.096 | -12.073 | 0.00 |
| PGC (%) | 50793745 | 2 | 7522822 | 0.000 | 0.056 | 0.112 | 13.681 | 0.00 |
| PGC (%) | 50815785 | 2 | 7544862 | 0.000 | 0.058 | 0.106 | 13.051 | 0.00 |
| PGC (%) | 50836797 | 2 | 7565874 | 0.001 | 0.225 | 0.099 | -6.840 | 0.00 |
| PGC (%) | 50925569 | 2 | 7654646 | 0.001 | 0.237 | 0.095 | -7.402 | 0.00 |
| PGC (%) | 50931518 | 2 | 7660595 | 0.001 | 0.179 | 0.093 | -7.310 | 0.00 |
| PGC (%) | 50970000 | 2 | 7699077 | 0.001 | 0.066 | 0.094 | -11.320 | 0.00 |
| PGC (%) | 89241523 | 3 | 10033350 | 0.000 | 0.306 | 0.099 | 6.873 | 0.00 |
| PGC (%) | 89249664 | 3 | 10041491 | 0.000 | 0.215 | 0.100 | -6.881 | 0.00 |
| PGC (%) | 89261170 | 3 | 10052997 | 0.001 | 0.136 | 0.094 | -7.993 | 0.00 |
| PGC (%) | 89266298 | 3 | 10058125 | 0.000 | 0.144 | 0.107 | -8.719 | 0.00 |
| PGC (%) | 89268449 | 3 | 10060276 | 0.001 | 0.136 | 0.094 | 7.993 | 0.00 |
| PGC (%) | 89270477 | 3 | 10062304 | 0.001 | 0.136 | 0.094 | -7.993 | 0.00 |
| PGC (%) | 102280155 | 3 | 23071982 | 0.001 | 0.326 | 0.096 | 7.189 | 0.00 |
| PGC (%) | 102309731 | 3 | 23101558 | 0.001 | 0.109 | 0.094 | -8.752 | 0.00 |
| PGC (%) | 102319129 | 3 | 23110956 | 0.000 | 0.167 | 0.099 | -8.063 | 0.00 |
| PGC (%) | 102334265 | 3 | 23126092 | 0.001 | 0.210 | 0.093 | 6.787 | 0.00 |
| PGC (%) | 102334320 | 3 | 23126147 | 0.000 | 0.172 | 0.100 | 7.926 | 0.00 |
| PGC (%) | 102337650 | 3 | 23129477 | 0.001 | 0.210 | 0.095 | -6.904 | 0.00 |
| PGC (%) | 102396192 | 3 | 23188019 | 0.001 | 0.086 | 0.096 | -9.854 | 0.00 |
| PGC (%) | 102436085 | 3 | 23227912 | 0.001 | 0.071 | 0.095 | -10.599 | 0.00 |
| PGC (%) | 102443580 | 3 | 23235407 | 0.001 | 0.422 | 0.097 | -6.575 | 0.00 |
|  |  |  |  |  |  |  |  |  |

**Table 3-2** QTLs mapping information of MLM analysis.

| **Sno.** | **QTL** | **SNP marker** | **P-Value** | **R^2^ of Model with SNP** | **Phenotypic variation** |  |
| --- | --- | --- | --- | --- | --- | --- |
|  |  |  |  |  |  |  |
| 1 | qAR_1.1 | S1_1085222 | 5.34E-04 | 0.07 | 0.00 |  |
| 2 | qAR_1.2 | S1_41079399 | 3.50E-04 | 0.08 | 0.00 |  |
| 3 | qAR_10.3 | S10_310793887 | 7.87E-06 | 0.12 | 0.02 |  |
| 4 | qAR_10.4 | S10_296092912 | 8.32E-05 | 0.09 | 0.00 |  |
| 5 | qAR_10.5 | S10_316603324 | 3.16E-05 | 0.07 | 0.03 |  |
| 6 | qAR_11.1 | S11_323072783 | 7.62E-05 | 0.09 | 0.00 |  |
| 7 | qAR_11.2 | S11_317050567 | 3.07E-05 | 0.07 | 0.03 |  |
| 8 | qAR_11.3 | S11_316963484 | 8.53E-04 | 0.07 | 0.00 |  |
| 9 | qAR_12.1 | S12_350264559 | 3.14E-05 | 0.07 | 0.80 |  |
| 10 | qAR_12.2 | S12_351239739 | 9.52E-04 | 0.07 | 0.00 |  |
| 11 | qAR_12.3 | S12_353433310 | 3.05E-05 | 0.07 | 0.00 |  |
| 12 | qAR_12.4 | S12_363008820 | 9.17E-04 | 0.07 | 0.00 |  |
| 13 | qAR_12.5 | S12_363123691 | 6.51E-04 | 0.07 | 0.02 |  |
| 14 | qAR_12.6 | S12_363180943 | 9.76E-05 | 0.09 | 0.00 |  |
| 15 | qAR_12.7 | S12_363658921 | 3.89E-04 | 0.08 | 0.00 |  |
| 16 | qAR_12.8 | S12_368046648 | 2.55E-04 | 0.08 | 0.06 |  |
| 17 | qAR_12.9 | S12_372943678 | 3.90E-04 | 0.08 | 0.004 |  |
| 18 | qAR_12.10 | S12_372990058 | 2.06E-04 | 0.08 | 0.07 |  |
| 19 | qAR_12.11 | S12_361505371 | 7.81E-04 | 0.07 | 0.00 |  |
| 20 | qAR_12.12 | S12_361676445 | 9.93E-04 | 0.07 | 0.00 |  |
| 21 | qAR_12.13 | S12_361891074 | 9.52E-05 | 0.09 | 0.00 |  |
| 22 | qAR_12.14 | S12_361917535 | 8.97E-06 | 0.12 | 0.00 |  |
| 23 | qAR_12.15 | S12_361994182 | 9.22E-04 | 0.07 | 0.00 |  |
| 1 | qHRR_1.1 | S1_4291505 | 7.96E-04 | 0.05 | 0.24 |  |
| 2 | qHRR_2.1 | S2_48834825 | 9.43E-04 | 0.05 | 0.00 |  |
| 3 | qHRR_3.1 | S3_107174613 | 8.44E-04 | 0.05 | 0.00 |  |
| 4 | qHRR_3.2 | S3_108852637 | 6.65E-04 | 0.05 | 0.00 |  |
| 5 | qHRR_4.1 | S4_146847512 | 8.00E-04 | 0.05 | 0.00 |  |
| 6 | qHRR_6.1 | S6_184750602 | 8.63E-06 | 0.10 | 0.00 |  |
| 7 | qHRR_6.2 | S6_184859992 | 5.58E-04 | 0.06 | 0.00 |  |
| 8 | qHRR_7.1 | S7_239100982 | 4.86E-04 | 0.06 | 0.07 |  |
| 9 | qHRR_7.2 | S7_240355009 | 5.88E-04 | 0.06 | 0.02 |  |
| 10 | qHRR_7.3 | S7_ 198210814 | 8.09E-04 | 0.05 | 0.02 |  |
| 11 | qHRR_8.1 | S8_246335910 | 5.90E-04 | 0.06 | 0.09 |  |
| 12 | qHRR_8.2 | S8_246596739 | 8.03E-04 | 0.05 | 0.41 |  |
| 13 | qHRR_8.3 | S8_258563043 | 5.96E-04 | 0.06 | 0.00 |  |
| 14 | qHRR_11.1 | S11_341143743 | 8.17E-04 | 0.05 | 0.00 |  |
| 15 | qHRR_11.2 | S11_344688985 | 2.21E-04 | 0.07 | 0.00 |  |
| 16 | qHRR_11.3 | S11_345564147 | 8.32E-04 | 0.05 | 0.00 |  |
| 17 | qHRR_11.4 | S11_343075408 | 9.87E-04 | 0.05 | 0.00 |  |
| 18 | qHRR_11.5 | S11_345603390 | 1.60E-04 | 0.07 | 0.09 |  |
| 19 | qHRR_11.6 | S11_336314352 | 7.48E-04 | 0.05 | 0.17 |  |
| 20 | qHRR_11.7 | S11_342574265 | 5.99E-05 | 0.08 | 0.00 |  |
| 21 | qHRR_12.1 | S12_367803774 | 8.20E-04 | 0.05 | 0.00 |  |
| 22 | qHRR_12.2 | S12_365173931 | 8.02E-04 | 0.05 | 0.00 |  |
| 1 | qPGC_1.1 | S1_14479845 | 8.89E-04 | 0.09 | 0.00 |  |
| 2 | qPGC_1.2 | S1_28603299 | 7.10E-04 | 0.10 | 0.00 |  |
| 3 | qPGC_10.1 | S10_308006255 | 8.00E-04 | 0.09 | 0.00 |  |
| 4 | qPGC_10.2 | S10_308329546 | 8.77E-04 | 0.09 | 0.00 |  |
| 5 | qPGC_11.1 | S11_332476062 | 7.60E-04 | 0.09 | 0.00 |  |
| 6 | qPGC_11.2 | S11_332935391 | 8.90E-04 | 0.09 | 0.00 |  |
| 7 | qPGC_11.3 | S11_333260269 | 3.35E-04 | 0.10 | 0.23 |  |
| 8 | qPGC_11.4 | S11_333763494 | 6.31E-05 | 0.12 | 0.28 |  |
| 9 | qPGC_11.5 | S11_333859080 | 7.02E-04 | 0.10 | 0.00 |  |
| 10 | qPGC_11.6 | S11_333874370 | 9.46E-06 | 0.14 | 0.00 |  |
| 11 | qPGC_11.7 | S11_334059073 | 8.13E-04 | 0.09 | 0.00 |  |
| 12 | qPGC_11.8 | S11_334259197 | 7.86E-04 | 0.09 | 0.00 |  |
| 13 | qPGC_11.9 | S11_345442853 | 6.14E-04 | 0.10 | 0.00 |  |
| 14 | qPGC_12.1 | S12_363057393 | 9.16E-04 | 0.09 | 0.00 |  |
| 15 | qPGC_12.2 | S12_365419932 | 9.39E-04 | 0.09 | 0.32 |  |
| 16 | qPGC_12.3 | S12_365438936 | 4.82E-04 | 0.10 | 0.00 |  |
| 17 | qPGC_12.4 | S12_348799363 | 9.76E-04 | 0.09 | 0.00 |  |
| 18 | qPGC_12.5 | S12_348841252 | 1.71E-01 | 0.02 | 0.03 |  |
| 19 | qPGC_2.1 | S2_54737512 | 9.41E-04 | 0.09 | 0.04 |  |
| 20 | qPGC_2.2 | S2_63940064 | 1.01E-04 | 0.11 | 0.00 |  |
| 21 | qPGC_2.3 | S2_64188701 | 8.62E-04 | 0.09 | 0.01 |  |
| 22 | qPGC_2.4 | S2_65195049 | 7.67E-04 | 0.09 | 0.00 |  |
| 23 | qPGC_2.5 | S2_67542265 | 8.09E-04 | 0.09 | 0.00 |  |
| 24 | qPGC_2.6 | S2_68352105 | 2.46E-05 | 0.13 | 0.00 |  |
| 25 | qPGC_2.7 | S2_77910183 | 7.14E-05 | 0.12 | 0.00 |  |
| 26 | qPGC_2.8 | S2_78445257 | 9.04E-05 | 0.12 | 0.00 |  |
| 27 | qPGC_2.9 | S2_50781811 | 9.08E-05 | 0.12 | 0.00 |  |
| 28 | qPGC_2.10 | S2_50931518 | 9.17E-04 | 0.09 | 0.00 |  |
| 29 | qPGC_3.1 | S3_89268449 | 8.25E-04 | 0.09 | 0.00 |  |
| 30 | qPGC_3.2 | S3_102334265 | 8.67E-04 | 0.09 | 0.00 |  |
| 31 | qPGC_3.3 | S3_102436085 | 7.26E-04 | 0.10 | 0.00 |  |
| 32 | qPGC_3.4 | S3_113672144 | 1.33E-01 | 0.02 | 0.00 |  |

**Supplementary Table 4:** The 75 published genes and their functional information of quality traits in rice.

| **Symbol** | **ID (MSU 7.0)** | **Chr** | | **Start** | | **End** | |
| --- | --- | --- | --- | --- | --- | --- | --- |
| MYB86-L1 | LOC_Os01g36460 | 1 | 20242740 | | 20240497 | |  |
| OsMADS2 | LOC_Os01g66030 | 1 | 38320785 | | 38324074 | |  |
| OsGA20ox-2 | LOC_Os01g66100 | 1 | 38382364 | | 38391782 | |  |
| OsNAC6 | LOC_Os01g66120 | 1 | 38398517 | | 38401533 | |  |
| OsbZIP04 | LOC_Os01g36220 | 1 | 20032340 | | 20035276 | |  |
| prx17 | LOC_Os01g36240 | 1 | 2006185 | | 20066601 | |  |
| OsNST1 | LOC_Os02g40030 | 2 | 24235668 | | 24239781 | |  |
| OsHCT2 | LOC_Os02g39850 | 2 | 24080704 | | 24084764 | |  |
| BIP135 | LOC_Os02g39920 | 2 | 24139753 | | 24146705 | |  |
| OsAGD2 | LOC_Os03g18810 | 3 | 10542847 | | 10546261 | |  |
| OsC3H21 | LOC_Os03g18950 | 3 | 10613535 | | 10615583 | |  |
| Hd1 | LOC_Os06g16370 | 6 | 9336359 | | 9338643 | |  |
| SEPK1 | LOC_Os06g16300 | 6 | 9289219 | | 9291362 | |  |
| OsATL4 | LOC_Os06g16420 | 6 | 9395957 | | 9400615 | |  |
| OsEnS-104 | LOC_Os07g20340 | 7 | 11738022 | | 11742263 | |  |
| PILS5 | LOC_Os07g04210 | 7 | 1845862 | | 1851637 | |  |
| OsC3H48 | LOC_Os07g04650 | 7 | 2062181 | | 2063847 | |  |
| BIP133 | LOC_Os07g04700 | 7 | 84106 | | 2091653 | |  |
| bZIP60 | LOC_Os07g44950 | 7 | 26808652 | | 26813801 | |  |
| OsSTA200 | LOC_Os07g45260 | 7 | 27006412 | | 27008541 | |  |
| OsTIFY5 | LOC_Os07g05830 | 7 | 2800810 | | 2804346 | |  |
| OsWRKY89 | LOC_Os08g17400 | 8 | 10633195 | | 10639603 | |  |
| OsHyPRP11 | LOC_Os10g20860 | 10 | 10570876 | | 10573989 | |  |
| OsHyPRP12 | LOC_Os10g20890 | 10 | 10587282 | | 10587977 | |  |
| OsRR22 | LOC_Os10g32600 | 10 | 17076098 | | 17077979 | |  |
| OsAMT1 | LOC_Os02g40730 | 2 | 24690884 | | 24692884 | |  |
| ONAC051 | LOC_Os02g41450 | 2 | 24816719 | | 24820281 | |  |
| OsG1L3 | LOC_Os02g41460 | 2 | 24823267 | | 24832173 | |  |
| OsRLCK113 | LOC_Os03g31260 | 3 | 17804694 | | 17805965 | |  |
| OsUFD2 | LOC_Os03g31400 | 3 | 17884893 | | 17892015 | |  |
| OsEXPA31 | LOC_Os03g31480 | 3 | 17934776 | | 17935904 | |  |
| OsFBX176 | LOC_Os05g49400 | 5 | 28327419 | | 28328921 | |  |
| OsFBX177 | LOC_Os05g49450 | 5 | 28350475 | | 28349165 | |  |
| DEGP9 | LOC_Os05g49380 | 5 | 28310905 | | 28318787 | |  |
| OsRpt5b | LOC_Os06g07630 | 6 | 3689672 | | 3694342 | |  |
| SRWD3 | LOC_Os06g07540 | 6 | 3632663 | | 3639659 | |  |
| OsFBX189 | LOC_Os06g07430 | 6 | 3571551 | | 3572234 | |  |
| OsUAM3 | LOC_Os07g41360 | 7 | 24773276 | | 24776526 | |  |
| OsMADS18 | LOC_Os07g41370 | 7 | 24788424 | | 24794187 | |  |
| Os_F0606 | LOC_Os07g45010 | 7 | 26851985 | | 26854751 | |  |
| OsPOP17 | LOC_Os07g48970 | 7 | 29303741 | | 29307647 | |  |
| OsFBDUF39 | LOC_Os07g48940 | 7 | 29282008 | | 29280094 | |  |
| OsNAS3 | LOC_Os07g48980 | 7 | 29323094 | | 29324723 | |  |
| OsALDH22 | LOC_Os07g48920 | 7 | 29271520 | | 29277745 | |  |
| Edf | LOC_Os08g27850 | 8 | 16982260 | | 16985504 | |  |
| ELF4_chr.8 | LOC_Os08g27860 | 8 | 16988231 | | 16995446 | |  |
| Osppc | LOC_Os08g27840 | 8 | 16964660 | | 16970614 | |  |
| OsBBX24 | LOC_Os08g08120 | 8 | 4608809 | | 4613096 | |  |
| OsMST5 | LOC_Os08g08070 | 8 | 4581432 | | 4583491 | |  |
| OsEMF2b | LOC_Os09g13630 | 9 | 7924338 | | 7938161 | |  |
| PFT1 | LOC_Os09g13610 | 9 | 7914083 | | 7925405 | |  |
| MT1a | LOC_Os11g47809 | 11 | 28827676 | | 28828513 | |  |
| Pik | LOC_Os11g46210 | 11 | 27983806 | | 27990549 | |  |
| OsFBDUF51 | LOC_Os11g36610 | 11 | 21609671 | | 21608163 | |  |
| EXPA26 | LOC_Os12g36040 | 12 | 22089434 | | 22091866 | |  |
| OsKCH1 | LOC_Os12g36100 | 12 | 22126680 | | 22134566 | |  |
| OsARF8 | LOC_Os02g41800 | 2 | 25133442 | | 25136111 | |  |
| OsPAL3 | LOC_Os02g41670 | 2 | 24992827 | | 24995423 | |  |
| OsPAL1 | LOC_Os02g41680 | 2 | 25006467 | | 25009211 | |  |
| OsCNGC3 | LOC_Os02g41710 | 2 | 25052086 | | 25055470 | |  |
| OsSERL4 | LOC_Os02g14120 | 2 | 7710439 | | 7716074 | |  |
| OsNAC32 | LOC_Os02g56600 | 3 | 34672992 | | 34676421 | |  |
| Emb2758 | LOC_Os02g06880 | 3 | 3468668 | | 34689898 | |  |
| OsWAK24 | LOC_Os02g56630 | 3 | 34720866 | | 34723359 | |  |
| OsRZ1 | LOC_Os03g61990 | 3 | 35134107 | | 35137345 | |  |
| GPA3 | LOC_Os03g61950 | 3 | 35115269 | | 35121803 | |  |
| OsFd3 | LOC_Os03g61960 | 3 | 35122440 | | 35124356 | |  |
| RAC3 | LOC_Os03g61970 | 3 | 35125518 | | 35129362 | |  |
| OsLTP1.3 | LOC_Os03g59380 | 3 | 33798938 | | 33800163 | |  |
| OsCESA2 | LOC_Os03g59340 | 3 | 33780013 | | 33786467 | |  |
| OsCPK8 | LOC_Os03g59390 | 3 | 33805800 | | 33810099 | |  |
| OsNAC65 | LOC_Os07g27330 | 7 | 15899431 | | 15902146 | |  |
| OsNAC102 | LOC_Os07g27340 | 7 | 15906886 | | 15908799 | |  |
| Urm1 | LOC_Os07g28280 | 7 | 16522444 | | 16524087 | |  |
| OsCDGSH | LOC_Os07g28400 | 7 | 16599911 | | 16602036 | |  |

**
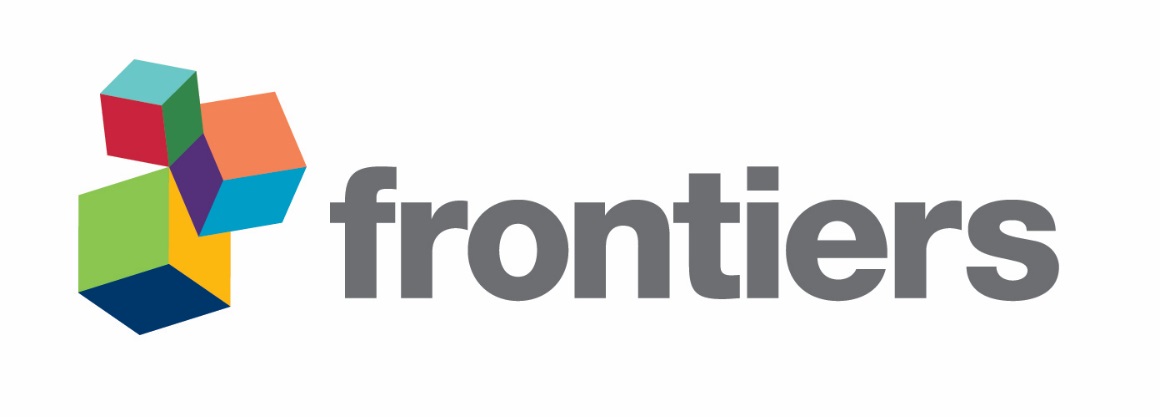
**

**Supplementary Figure 1.** Distribution of quality related grain QTNs identified using the three multi-locus GWAS models. (A) Number of significant QTNs detected for 3 quality traits across 3 multi-locus GWAS methods. (B) Number of significant QTNs detected using each of 3 multi-locus GWAS methods.

**Supplementary Table 1:** Details of 196 rice accessions used in the present study.

**Supplementary Table 2:** Descriptive statistics for three grain quality traits of rice.

**Supplementary Table 3:** SNPs and QTLs mapping information in MLM analysis.

**Supplementary Table 4:** The 75 published genes and their functional information of quality traits in rice.
